# Migration through physical constraints is enabled by MAPK-induced cell softening via actin cytoskeleton re-organization

**DOI:** 10.1242/jcs.224071

**Published:** 2019-05-31

**Authors:** Dominika A. Rudzka, Giulia Spennati, David J. McGarry, Ya-Hua Chim, Matthew Neilson, Aleksandra Ptak, June Munro, Gabriela Kalna, Ann Hedley, Daniela Moralli, Catherine Green, Susan Mason, Karen Blyth, Margaret Mullin, Huabing Yin, Michael F. Olson

**Affiliations:** 1 Cancer Research UK Beatson Institute, Garscube Estate, Switchback Road, Glasgow G61 1BD, UK; 2 School of Engineering, University of Glasgow, Glasgow G12 8QQ, UK; 3 Wellcome Trust Centre for Human Genetics, University of Oxford, Roosevelt Drive, Oxford OX3 7BN, UK; 4 Electron Microscopy Facility, Department of Chemistry, University of Glasgow, Glasgow G12 8QQ, UK; 5 Institute of Cancer Sciences, University of Glasgow, Glasgow G12 8QQ, UK

**Keywords:** MAPK, Cytoskeleton, Elasticity, Motility

## Abstract

Cancer cells are softer than the normal cells, and metastatic cells are even softer. These changes in biomechanical properties contribute to cancer progression by facilitating cell movement through physically constraining environments. To identify properties that enabled passage through physical constraints, cells that were more efficient at moving through narrow membrane micropores were selected from established cell lines. By examining micropore-selected human MDA MB 231 breast cancer and MDA MB 435 melanoma cancer cells, membrane fluidity and nuclear elasticity were excluded as primary contributors. Instead, reduced actin cytoskeleton anisotropy, focal adhesion density and cell stiffness were characteristics associated with efficient passage through constraints. By comparing transcriptomic profiles between the parental and selected populations, increased Ras/MAPK signalling was linked with cytoskeleton rearrangements and cell softening. MEK inhibitor treatment reversed the transcriptional, cytoskeleton, focal adhesion and elasticity changes. Conversely, expression of oncogenic KRas in parental MDA MB 231 cells, or oncogenic BRaf in parental MDA MB 435 cells, significantly reduced cell stiffness. These results reveal that MAPK signalling, in addition to tumour cell proliferation, has a significant role in regulating cell biomechanics.

This article has an associated First Person interview with the first author of the paper.

## Abstract (precis)

**Highlighted Article:** Selection for tumour cells that efficiently pass through narrow diameter microporous membranes reveals a prominent role for MAPK signalling in regulating cell elasticity.

## INTRODUCTION

The metastatic spread of cancer cells from primary tumours to distant sites is the leading cause of cancer mortality, contributing to as much as 90% of cancer-related deaths (Chaffer and Weinberg, 2011; Sporn, 1996). The life-threatening aspects of some cancers, including breast cancers and melanoma, derive from the harm caused at distant essential sites, including lung, liver, bones and brain, rather than at their tissue of origin (Luke et al., 2017; Melzer et al., 2017).

Tumour cells passing through barriers (e.g. basement membrane, tumour stroma and endothelial layers) encountered during metastasis experience varying forms of physical confinement. Spaces in three-dimensional (3D) extracellular matrix (ECM) create pores or tunnel-like tracks, which often may be smaller than metastasizing cells (Doerschuk et al., 1993; Weigelin et al., 2012). These pores and tracks exist in healthy tissues; for example, they occur along or within blood vessels (Lugassy et al., 2014), between muscle and nerve fibres (Weigelin et al., 2012), between collagen fibres (Gritsenko et al., 2012), or within brain perivascular spaces (Charles and Holland, 2010). Gaps may also be created or expanded by tumour-associated stromal cells, which play active roles in enabling tumour cell spread (Erdogan and Webb, 2017; Nielsen and Schmid, 2017). Processes that increase aperture size can facilitate cell passage through narrow gaps. For example, ECM degradation by enzymes, such as matrix metalloproteases, is a common mechanism to enable tumour cell invasion (Paterson and Courtneidge, 2018). Physical force generated by migrating cells can expand pores to allow passage (Chang et al., 2017). However, these processes may not be appropriate in all contexts, or may be inadequate to increase pore size sufficiently to enable efficient movement through confined environments. As a result, cell pliability to allow changes in shape that enable a cell to squeeze through narrow physical constraints can also play a considerable role in local tissue invasion and consequent metastasis (Alibert et al., 2017).

Cancer cells are heterogeneous for many properties (Altschuler and Wu, 2010), this diversity engenders adaptability to environmental conditions and contributes to disease progression (Meacham and Morrison, 2013). Although typically isolated as individual clones from patient tumours, single-cell analytical approaches including transcriptional analysis, mapping of chromatin organization and high-content imaging (Litzenburger et al., 2017; Yin et al., 2013; Zamanighomi et al., 2018), have revealed that, although they may be homogenous for their underlying genetic alterations, established tumour cell lines are actually heterogeneous for numerous characteristics and behaviours. The imposition of selective pressures can result in positive selection for advantageous properties from dispersed distributions of specific parameters.

To identify, in an unbiased manner, the properties that enable migration through confined spaces, MDA MB 231 human breast cancer cells were selected (denoted Sel1, Sel2 etc.) from starting parent (denoted Parent) cell populations by multiple rounds of passage through 3-μm-diameter microporous membranes that severely impede migration. The MDA MB 231 Parent and pore-selected cells were then used for two comparisons. Firstly, by relating the characteristics of Parent and pore-selected cells to cells selected by flow sorting for small size, it was possible to determine which properties were specifically associated with passage through small spaces, and eliminate those characteristics that were linked specifically with small cell size. Secondly, by comparing those properties that differed between Parent and pore-selected MDA MB 231 breast cancer cells with those that also differed between Parent and pore-selected MDA MB 435 melanoma cells, shared factors that enabled movement through narrow gaps were determined. Through these comparisons, we concluded that although pore-selected cells were small, size was not a primary determinant of confined migration, nor were nuclei size or elasticity, which were linked with cell size but not the ability to pass through small diameter pores. Instead, decreased filamentous actin (F-actin) anisotropy, focal adhesion density and cell stiffness were defining characteristics of pore-selected cells. Transcriptomic analysis identified increased Ras/mitogen-activated protein kinase (MAPK) signalling output in pore-selected cells, while inhibition of the activity of the upstream activators of the MAPKs ERK1 and ERK2 (ERK1/2, also known as MAPK3 and MAPK1, respectively), the MEK1 and MEK2 proteins (hereafter MEK1/2, also known as MAP2K1 and MAP2K2), reversed the decreased F-actin anisotropy, focal adhesion density and cell stiffness. Increased MAPK signalling in unselected Parental cells was sufficient to decrease cell stiffness, indicating that Ras/MAPK signalling plays a significant role in regulating cell biomechanical properties, in addition to its well characterized and central contributions to proliferation. These findings suggest that MAPK signalling output might contribute widely to cancer metastasis, and that inhibition of MAPK signalling could have broad clinical benefits by increasing metastatic cell stiffness to restrict movement through confined environments.

## RESULTS

### Pore-selected cells have properties that enable invasion

**Fig. 1.**
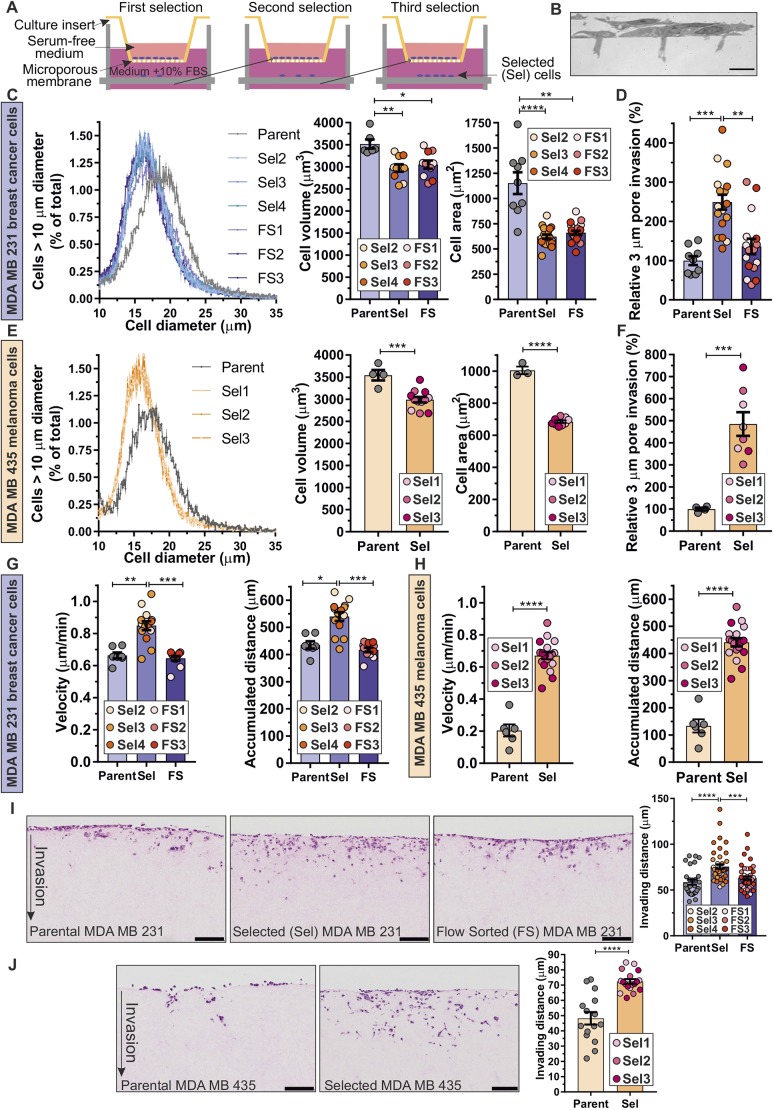
**Small-diameter-pore selection enriches for invasive and metastatic cell behaviours.** (A) Schematic diagram of pore-selection strategy. Cells (10^6^) were placed in serum-free medium in 7.5 cm diameter tissue culture inserts with microporous membranes (3 µm pore diameter), and allowed to migrate for 5 days towards serum-containing medium below. ‘Successful’ cells were collected, expanded and re-plated twice more as above. Three separate pore-selected (Sel) populations were isolated for each cell line. (B) Transmission electron micrograph showing MDA MB 231 cells on the membrane surface and extending downwards into 3 µm pores. Scale bar: 10 µm. (C) Left graph, MDA MB 231 Parent, three independent pore-selected (Sel2, Sel3, Sel4) and three independent flow-sorted (FS1, FS2, FS3) mean±s.d. cell diameter profiles for cells greater than 10 µm at 0.1 µm bins from three replicates. Middle and right graphs, mean±s.e.m. cell volumes (Parent, *n*=6; Sel and FS, *n*=10 replicates) and areas (Parent, *n*=9; Sel or FS, *n*=20–21 replicates). *P<0.05, **P<0.01, ****P<0.0001 (Kruskal–Wallis one-way ANOVA and Dunn's multiple comparisons test). (D) MDA MB 231 Parent, Sel and FS relative (%, mean±s.e.m.) cell migration through 3 µm pore inserts was assessed after 3 days (Parent, *n*=9; Sel and FS, *n*=16–18 replicates). **P<0.01, ***P<0.001 (Kruskal–Wallis one-way ANOVA and Dunn's multiple comparisons test). (E) Left graph, MDA MB 435 Parent and three independent pore-selected (Sel1, Sel2, Sel3) mean±s.d. cell diameter profiles for cells greater than 10 µm at 0.1 bins from three replicates. Middle and right graphs, mean±s.e.m. cell volumes (Parent, *n*=4; Sel, *n*=12 replicates) and areas (Parent, *n*=3; Sel, *n*=9 replicates). ***P<0.001, ****P<0.0001 (Student's *t*-test). (F) MDA MB 435 Parent and Sel relative (%, mean±s.e.m.) cell migration through 3 µm pore inserts was assessed after 3 days (left; Parent *n*=4, Sel *n*=8 replicates). ***P<0.001 (Student's *t*-test). (G) MDA MB 231 Parent, Sel and FS single-cell random migration was tracked for 22 h. Left graph; mean±s.e.m. velocities. Right graph, mean±s.e.m. accumulated distance. Parent, *n*=7; Sel, *n*=14; FS, *n*=11. *P<0.05, **P<0.01, ***P<0.001 (Kruskal–Wallis one-way ANOVA and Dunn's multiple comparisons test). (H) MDA MB 435 Parent and Sel single-cell random migration was tracked for 22 h. Left graph, mean±s.e.m. velocities. Right graph, mean±s.e.m. accumulated distance. Parent, *n*=6; Sel, *n*=18. ****P<0.0001 (Student's *t*-test). (I) Single-cell invasion into fibroblast-conditioned collagen matrices for MDA MB 231 cells. Scale bars: 100 µm. Individual cell distances invaded from the upper collagen surfaces were measured, and mean±s.e.m. distances from independent determinations were plotted (Parent, *n*=28; Sel, *n*=45; FS, *n*=45 replicates). ***P<0.001, ****P<0.0001 (one-way ANOVA and Tukey's multiple comparisons test). (J) Single-cell invasion distances into fibroblast-conditioned collagen matrices for MDA MB 435 cells. Scale bars: 100 µm. Mean±s.e.m. cell distances invaded from independent determinations were plotted (Parent, *n*=15; Sel, *n*=18 replicates). ****P<0.0001 (Student's *t*-test).

To isolate cells with properties that enable passage through narrow physical constraints, cells were plated in serum-free medium in tissue culture inserts with microporous membranes (3 µm average diameter pores) that severely restrict cell movement (Fig. 1A,B). By collecting cells that had passed through the physical constraints to the serum-containing medium below, and subjecting them to two additional rounds of selection, three independent populations of pore-selected (Sel2, Sel3 and Sel4) MDA MB 231 D3H2LN luciferase (abbreviated MDA MB 231; Jenkins et al., 2005) breast cancer, and MDA MB 435 melanoma (Sel1, Sel2 and Sel3), cells were isolated. For comparison purposes, three rounds of flow sorting were used to isolate three independent populations (FS1, FS2 and FS3) of small diameter MDA MB 231 cells (Fig. S1A). The distribution of suspended Sel or FS cell diameters were similarly shifted towards smaller sizes relative to Parent cells (Fig. 1C, left panel), which resulted in significantly smaller (by 15%) mean cell volumes (Fig. 1C, middle panel; Fig. S1B). Single-cell high-content imaging revealed that the two-dimensional (2D) areas of Sel and FS isolates were both significantly smaller (by ∼45%) than Parent MDA MB 231 cells (Fig. 1C, right panel; Fig. S1C). However, the pore-selected Sel cells were significantly more efficient (greater than two times) at migrating through 3 µm pore tissue culture inserts than Parent or FS populations (Fig. 1D). Similarly, the diameters of three independent populations (Sel1, Sel2 and Sel3) of pore-selected MDA MB 435 cells were shifted towards a smaller distribution relative to Parent cells (Fig. 1E, left panel), which resulted in significantly smaller 3D volumes (by ∼15%) than Parent cells (Fig. 1E, middle panel; Fig. S1D). In addition, high-content imaging revealed that Sel populations had a ∼30% reduction in 2D area relative to Parent MDA MB 435 cells (Fig. 1E, right panel; Fig. S1E). Similar to the pore-selected MDA MB 231 cells, the MDA MB 435 Sel isolates were significantly more efficient (∼4.5 times) at moving through 3 µm pore tissue culture inserts compared to Parent cells (Fig. 1F).

Single-cell random migration was tracked for the Parent, Sel and FS populations over a 22 h period, which revealed that pore-selected MDA MB 231 cells were ∼28% faster (Fig. 1G, left panel; Fig. S2A,B), and travelled ∼24% further (Fig. 1G, right panel; Fig. S2C) than Parent or FS populations. Similarly, pore-selected MDA MB 435 cells were ∼3.3 times faster (Fig. 1H, left panel; Fig. S2D,E) and travelled ∼3.4 times further (Fig. 1H, left panel; Fig. S2F) than Parent cells.

To determine whether the properties that facilitate migration through narrow physical constraints would also contribute to 3D extracellular matrix (ECM) invasion, the distances that individual cells invaded through dense fibroblast-remodelled collagen matrices towards serum-containing medium were determined (Rath et al., 2017). The MDA MB 231 Sel isolates invaded significantly further (by 25%) into collagen matrices than either Parent or FS populations (Fig. 1I; Fig. S2G), which was paralleled by the 50% greater distances invaded by Sel MDA MB 435 isolates compared to Parent cells (Fig. 1J; Fig. S2H). Importantly, proliferation of all selected populations did not differ significantly relative to the starting Parent MDA MB 231 (Fig. S1F) or Parent MDA MB 435 (Fig. S1G), indicating that the differences in invasion were not influenced by cell number. These results indicate that cells with properties that enable migration through narrow gaps in synthetic membranes, as well as through dense collagen matrices, can be isolated from established tumour cell lines.

### Nuclear and membrane properties do not contribute to confined migration

**Fig. 2.**
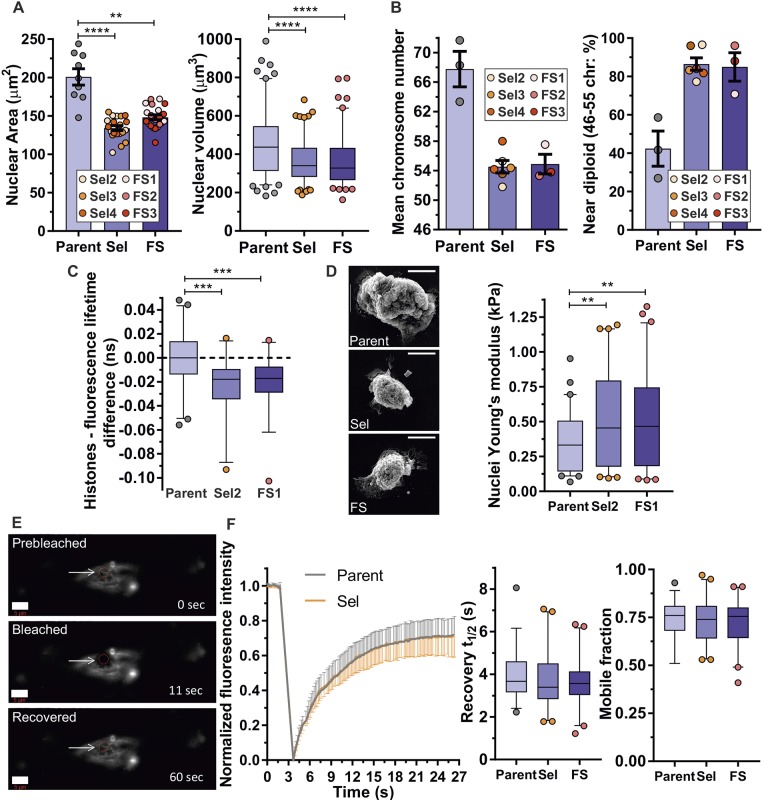
**Pore migration is not enabled by nuclear or membrane properties.** (A) Parent, Sel and FS MDA MB 231 mean±s.e.m. nuclear areas (left; Parent *n*=9, Sel and FS, *n*=20–21 replicates) and nuclear volumes (right; Parent, *n*=120; Sel and FS, *n*=111–123;). **P<0.01, ****P<0.0001 (left, Kruskal–Wallis one-way ANOVA and Dunn's multiple comparisons test; right, one-way ANOVA and Tukey's multiple comparisons test). (B) Cytogenetic determination (minimum 20 cells per experiment) of mean±s.e.m. chromosome number (left; Parent, *n*=3; Sel and FS, *n*=3–6 replicates) and percentage of cells near diploidy (right; 46–55 chromosomes). (C) Differences in FRET fluorescence lifetimes for nuclei labelled with GFP–H2B and RFP–H2B for Parent (*n*=40), Sel2 (*n*=38) and FS1 (*n*=38) MDA MB 231 cells. ***P<0.001 (Kruskal–Wallis one-way ANOVA and Dunn's multiple comparisons test). (D) Scanning electron micrographs of isolated nuclei from Parent, Sel and FS MDA MB 231 cells. Scale bars: 5 µm. The Young's modulus was determined by AFM for isolated nuclei from Parent (*n*=60), Sel2 (*n*=61) and FS1 (*n*=71) MDA MB 231 cells. **P<0.01 (one-way ANOVA and Tukey's multiple comparisons test). (E) FRAP of NBD-C6-sphingomyelin in 3 µm diameter regions at the indicated times. The arrows points to the bleached region. Scale bar: 5 µm. (F) FRAP traces (left) indicating normalized fluorescence intensity for Parent (*n*=12 cells) and Sel2 (*n*=7 cells) MDA MB 231 cells at the indicated times. Times for 50% recovery (*t*_1/2_; centre graph; Parent, *n*=39, Sel and FS, *n*=42–43) and mobile fraction (right graph). For all box plots, the box represents the 25–75th percentiles, and the median is indicated. The whiskers show the 5–95th percentiles, and outliers are indicated.

The nucleus, as the largest and stiffest organelle, has considerable influence on cell migration through confined environments (reviewed in Charras and Sahai, 2014). High-content imaging revealed that the 2D nuclear areas of MDA MB 231 Sel and FS populations were both similarly smaller (by ∼25%) than those for Parent cells (Fig. 2A, left panel; Fig. S3A), while the volumes of individual isolated Sel and FS nuclei were equivalently reduced by ∼25% (Fig. 2A, right panel; Fig. S3B). To characterize possible contributors to smaller nuclei in Sel and FS populations, chromosomes were counted by cytogenetic methods. Consistent with previous reports (Crepin et al., 1990), Parent MDA MB 231 cells were hyperploid, with ∼68 chromosomes per cell (Fig. 2B, left; Fig. S3C). In contrast, Sel and FS populations had ∼54 chromosomes per cell, corresponding to >80% of cells being near diploid (46–55 chromosomes), compared to only ∼40% for Parental cells (Fig. 2B, right; Fig. S3D,E). Chromatin compaction in Parent, Sel2 and FS1 populations were compared by using a fluorescence lifetime imaging-fluorescence resonance energy transfer (FLIM-FRET)-based method, in which energy transfer between histone H2B proteins fused to GFP (donor fluorophore) or mCherry (acceptor fluorophore), which decreases GFP fluorescence lifetime, occurs when the proteins are within ∼10 nm of each other (Llères et al., 2009). Chromatin compaction changes were first validated in Parent and Sel2 MDA MB 231 cells following 2-deoxyglucose (2-DG) plus NaN_3_-induced ATP depletion (Visvanathan et al., 2013) (Fig. S3F,G). Relative to the mean fluorescence lifetimes determined for Parent MDA MB 231 cells, representative Sel2 and FS1 isolates had similarly decreased fluorescence lifetimes, indicating comparable chromatin compaction (Fig. 2C). Since nuclear mechanical properties, which have previously been shown to affect migration in confined environments (McGregor et al., 2016), are influenced by chromatin compaction (Furusawa et al., 2015), the elasticities (Young's modulus) of isolated nuclei from Parent, Sel2 and FS1 populations (Fig. 2D, left panel) were measured by atomic force microscopy (AFM) (McPhee et al., 2010). The elasticities of Sel2 and FS1 nuclei were significantly increased relative to Parent MDA MB 231 nuclei (Fig. 2D, right panel). These results show that nuclear size and mechanical properties, associated with changes in chromosome number and compaction, are not the dominant factors that enable pore-selected cells to migrate more efficiently through narrow gaps. Nuclear size and DNA content were instead directly proportional to cell size, in agreement with previous observations (Gillooly et al., 2015; Levy and Heald, 2012).

Changes in membrane fluidity, due to factors including membrane composition and microdomain organization, have been reported to be associated with altered cancer cell migration (Edmond et al., 2015; Sade et al., 2012; Zhao et al., 2016). To determine whether there were differences in membrane fluidity that might enable pore-selected cells to move more effectively in confined environments, fluorescence recovery after photobleaching (FRAP) analysis (Fig. 2E) was performed with MDA MB 231 Parent, Sel2 and FS1 isolates. By measuring the lateral diffusion of NBD-C6-sphingomyelin into a discrete 3 µm diameter photobleached regions (Fig. 2F, left panel), no significant differences in the time for 50% recovery (*t*_1/2_; Fig. 2F, centre panel; Fig. S3H) or the maximum extent of recovery (mobile fraction; Fig. 2F, right panel; Fig. S3I) between the Parent, Sel2 or FS1 populations were detected, indicating that altered membrane fluidity was not a property of cells selected through pores or by flow sorting.

### Pore-selected cells have altered cytoskeleton organization and focal adhesion density, which is associated with decreased stiffness

**Fig. 3.**
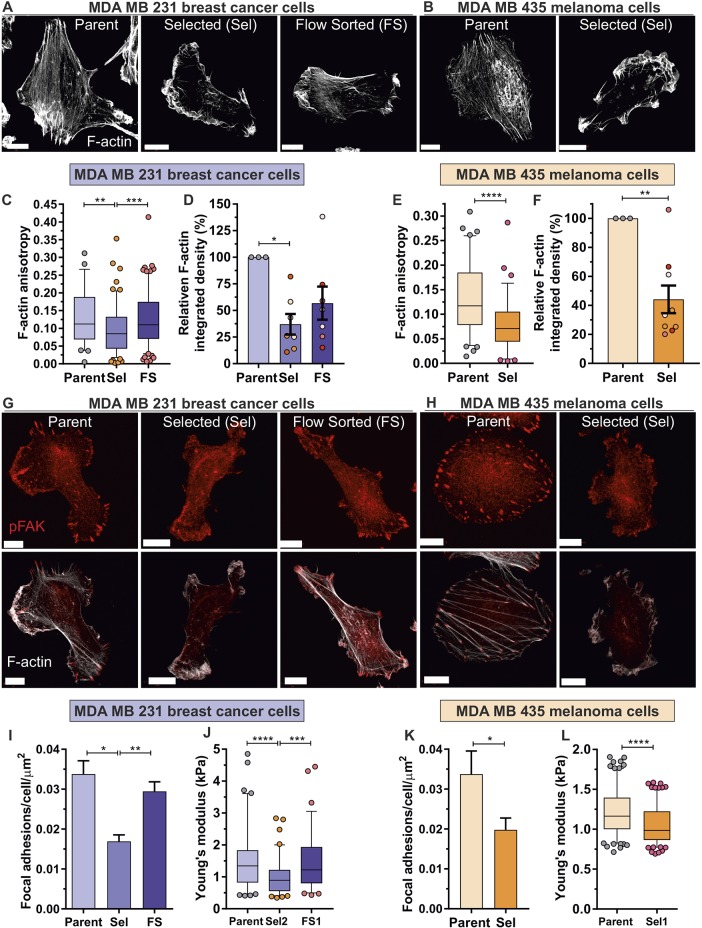
**Decreased F-actin anisotropy and intensity, focal adhesion density and Young's modulus in pore-selected cells.** (A) Parent, Sel and FS MDA MB 231 cells were fixed and stained with phalloidin for F-actin. Scale bars: 5 µm. (B) Parent and Sel MDA MB 435 cells were fixed and stained for F-actin. Scale bars: 5 µm. (C) TIRF images of phalloidin-stained Parent, Sel and FS MDA MB 231 cells were scored for F-actin anisotropy. Parent, *n*=59, Sel and FS, *n*=138–145. **P<0.01, ***P<0.001 (one-way ANOVA and Tukey's multiple comparisons test). (D) Mean±s.e.m. relative F-actin integrated density normalized to Parent levels (set at 100%). *P<0.05 (Kruskal–Wallis one-way ANOVA and Dunn's multiple comparisons test). (E) TIRF images of phalloidin-stained Parent and Sel FS MDA MB 435 cells were scored for F-actin anisotropy. Parent, *n*=88; Sel, *n*=78. ****P<0.0001 (Student's *t*-test). (F) Mean±s.e.m. relative F-actin integrated density normalized to Parent levels (set at 100%). **P<0.01 (Student's *t*-test). (G) Parent, Sel and FS MDA MB 231 cells were fixed and stained for pFAK to mark focal adhesions (upper panels) and with phalloidin for F-actin (lower panel, overlay). Scale bars: 5 µm. (H) Parent and Sel MDA MB 435 cells were fixed and stained for pFAK to mark focal adhesions (upper panels) and with phalloidin for F-actin (lower panel, overlay). Scale bars: 5 µm. (I) Mean±s.e.m. focal adhesion density (Parent, *n*=3, Sel and FS, *n*=9 replicates). *P<0.05, **P<0.01, ***P<0.001 (Kruskal–Wallis one-way ANOVA and Dunn's multiple comparisons test). (J) Cell elasticity (Young's modulus) of Parent (*n*=87), Sel2 (*n*=88) and FS1 (*n*=79) MDA MB 231 cells. ***P<0.001, ****P<0.0001 (one-way ANOVA and Tukey's multiple comparisons test). (K) Mean±s.e.m. focal adhesion density (Parent, *n*=4; Sel, *n*=6 replicates). *P<0.05 (Student's *t*-test). (L) Young's modulus of Parent (*n*=160) and Sel1 (*n*=160) MDA MB 435 cells. ****P<0.0001 (Student's *t*-test). For all box plots, the box represents the 25–75th percentiles, and the median is indicated. The whiskers show the 5–95th percentiles, and outliers are indicated.

The organization of the F-actin cytoskeleton is a major factor regulating cancer cell migration (Olson and Sahai, 2009) and determining cell mechanical properties, with F-actin fibre organization and focal adhesions both contributing to cell stiffness (Gupta et al., 2016, 2015; McPhee et al., 2010). High-magnification imaging of phalloidin-stained fixed cells revealed obvious differences in F-actin structures, with pore-selected Sel MDA MB 231 cells (Fig. 3A) and Sel MDA MB 435 cells (Fig. 3B) having fewer long and parallel filaments. To quantify these differences, total internal reflectance microscopy (TIRF) was used to reveal F-actin structures along a ∼0.2 µm plane at cell–substrate interfaces (Fig. S4A,B), to eliminate possible effects of cell height differences. The organization of F-actin filaments can be described as isotropic, when there is a uniform distribution in all directions, or anisotropic, if the organization is directional. The degree of F-actin bundling and cross-linking into elongated structures, such as stress fibres, which are typically parallel in orientation, can be quantified as an increase in actin anisotropy. Importantly, actin filament cross-linking increases cytoskeleton stiffness (Rajagopal et al., 2018); therefore, F-actin anisotropy is proportional to stiffness. TIRF images were analysed for F-actin anisotropy (Boudaoud et al., 2014), which revealed that Sel MDA MB 231 isolates had a significantly reduced F-actin anisotropy relative to Parent or FS populations (Fig. 3C). In addition, these TIRF images were quantified for relative F-actin integrated density, which takes into account fluorescence intensity and cell area to reflect total F-actin levels, revealing that MDA MB 231 Sel populations had significantly lower total F-actin that Parent cells (Fig. 3D). Similar results were observed for Sel MDA MB 435 cells, with significantly lower F-actin anisotropy (Fig. 3E) and total F-actin levels (Fig. 3F).

Focal adhesions communicate external force inwards to modify cytoskeleton structures, and are responsive to internal cytoskeleton-derived contractile force (Bershadsky et al., 2003; Romer et al., 2006). Since all cells were plated on identical substrates, focal adhesion density is an independent indicator of cytoskeleton organization. Accompanying these F-actin changes, there were lower densities of focal adhesions as detected by immunofluorescence imaging of phosphorylated focal adhesion kinase (pFAK; FAK is also known as PTK2) in Sel MDA MB 231 cells (Fig. 3G, upper panels) and in Sel MDA MB 435 cells (Fig. 3H, upper panels). Focal adhesions per cell were corrected for cell area to generate focal adhesion density scores, which showed that Sel MDA MB 231 isolates had significantly lower focal adhesion densities (by ∼55%) relative to Parent or FS populations (Fig. 3I; Fig. S4C).

The contribution of the F-actin cytoskeleton to cell stiffness was validated for MDA MB 231 cells with the actin polymerization inhibitor Cytochalasin D, which disrupted F-actin and decreased cells stiffness as determined by AFM (Fig. S4E). The elasticities (Young's modulus) of Sel MDA MB 231 cells were significantly less stiff than Parent or FS populations (Fig. 3J). Similar to the reduced F-actin anisotropy (Fig. 3E) and total F-actin (Fig. 3F) in Sel MDA MB 435 cells relative to Parent cells, there also were significantly lower numbers of focal adhesion densities (by ∼40%) (Fig. 3K; Fig. S4D) and significantly reduced cell stiffness (Fig. 3L). These results demonstrate that F-actin organization and levels, focal adhesion density and cell elasticity are inter-related properties associated with the ability of pore-selected cells to efficiently move through confined environments.

### Pore-selected cells have elevated MAPK signalling as revealed by RNA sequencing

**Fig. 4.**
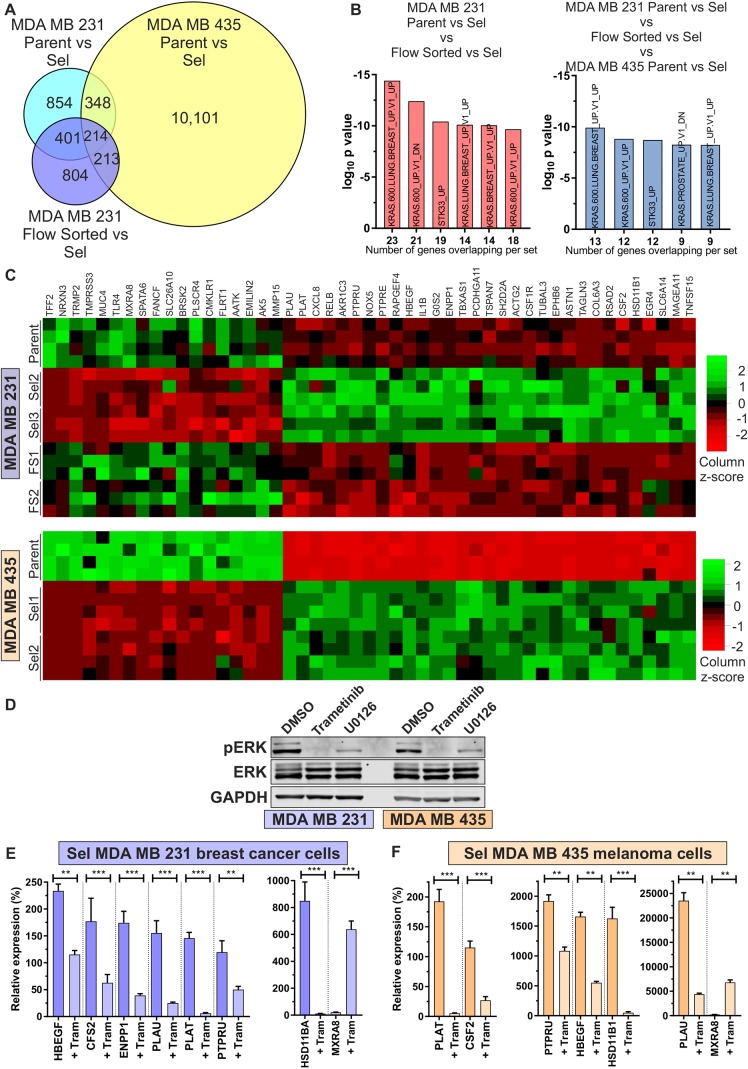
**RNA sequencing reveals increased Ras/MAPK signal output in pore-selected cells.** (A) Venn diagram indicating number of shared changes in gene expression (FC>1.5×; *P*<0.05) for MDA MB 231 Parent versus Sel (light blue; 1817), MDA MB 231 FS versus Sel (dark blue; 1632) and MDA MB 435 Parent versus Sel (yellow; 10,876). The gene set associated with pore selection, and not small size [(Parent versus Sel) versus (FS versus Sel)] for MDA MB 231 comprised 615 genes, of which 214 were also altered in MDA MB 435 Parent versus Sel. (B) The most-significant GSEA ‘oncogenic signatures’ identified for the MDA MB 231 615 gene set (left) or 214 combined gene set (right). (C) Relative expression of 49 genes from the 214 combined gene set that were identified in GSEA RAS/RAF/MEK-related ‘oncogenic signatures’. *Z*-scores were calculated from number of RNA sequence reads in the experimental replicates of Parent, Sel2, Sel3, FS1, FS2 MDA MB 231 cells (upper panel) within the column for each gene. Similarly, gene expression was compared by calculating *Z*-scores from the RNA sequences reads in the experimental replicates for Parent, Sel1 and Sel2 MDA MB 435 cells (lower panel). Green indicates relatively higher expression, red indicates relatively lower expression. (D) Western blots of phosphorylated active ERK1/2 (pERK) and total ERK1/2 (ERK) for MDA MB 231 Sel2 and MDA MB 435 Sel1 populations following treatment with DMSO vehicle, 0.5 µM Trametinib or 10 µM U0126 for 18 h. GAPDH is shown as a loading control. (E) Mean±s.e.m. gene expression levels in the indicated Sel MDA MB 231 cells were determined by qPCR relative to Parent cells (set at 100%) following treatment with vehicle DMSO or 0.5 µM Trametinib (Tram) for 24 h. **P<0.01, ***P<0.001 between DMSO and Tram for each gene (Student's *t*-test; *n*=9 replicates). (F) Mean±s.e.m. relative gene expression in indicated Sel MDA MB 435 cells following treatment with DMSO or 0.5 µM Trametinib (Tram) for 24 h. **P<0.01, ***P<0.001 (Student's *t*-test; *n*=6–9 replicates).

Given that the properties of pore-selected and flow-sorted cells that differentiated them from Parent MDA MB 231 or MDA MB 435 cells were stably maintained over time and multiple cell passages, their differences in constrained migration abilities could be due to stable transcriptional differences between populations. RNA sequencing (RNA-seq) was performed on polyA^+^-enriched RNA from Parent MDA MB 231 cells, two pore-selected isolates (Sel2, Sel3) and two flow-sorted isolates (FS1, FS2), as well as parental MDA MB 435 cells and two pore-selected isolates (Sel1, Sel2), as described in Rudzka et al. (2017). By comparing mRNA transcript reads of Parent MDA MB 231 with pore-selected Sel1 and Sel2 cells, using a ±1.5 fold-change (FC) cut-off and a statistical threshold of *P*<0.05, 1817 genes were differentially expressed (Fig. 4A). Comparing pore-selected MDA MB 231 Sel1 and Sel2 cells with flow-sorted FS1 and FS2 cells revealed 1632 gene expression differences (Fig. 4A). The overlap between the two comparisons was comprised of 615 genes, which were independent of differing cell and nucleus size but associated with constrained migration ability (Fig. 4A). Using the same criteria of a ±1.5 FC cut-off and statistical threshold of *P*<0.05, the number and magnitude of gene expression differences between Parent and two pore-selected Sel1 and Sel2 MDA MB 435 populations were even greater, with 10,876 differentially expressed genes (Fig. 4A). When compared with the patterns of gene expression in MDA MB 231 Parent and Sel populations, there were 214 genes that were consistently associated with constrained migration ability (Fig. 4A), which we define as the pore-invasion gene set. When the 615 gene set associated with pore-invasion from MDA MB 231 cells was compared with 189 ‘Oncogenic Signature’ gene sets using Gene Set Enrichment Analysis (GSEA) (Subramanian et al., 2005), five of the six most statistically significant gene sets were associated with oncogenic KRas signalling (Fig. 4B, left panel). Similarly, when the 214 pore-invasion gene set common to MDA MB 231 and MDA MB 435 cells was analysed by GSEA, five of the six most statistically significant oncogenic signature gene sets were associated with KRas signalling (Fig. 4B, right panel). In total, 49 individual genes from the 214 pore-invasion gene set were associated with statistically significant KRas, BRaf and/or MAPK-linked oncogenic signature gene sets, of which 18 were expressed at lower levels and 31 at higher levels in Sel2 and Sel3 populations relative to Parent and flow-sorted FS1 and FS2 MDA MB 231 cells (Fig. 4C, upper panel). The same patterns of up- and down-regulated gene expression were observed when comparing pore-selected Sel1 and Sel2 isolates with Parent MDA MB 435 cells (Fig. 4C, lower panel). We verified that signalling via the MAPK pathway could blocked by two structurally unrelated MEK inhibitors with differing mechanisms of action, the allosteric MEK1/2 inhibitor Trametinib (Grimaldi et al., 2017; Roskoski, 2017) and the non-competitive inhibitor U0126 (Favata et al., 1998), in both MDA MB 231 and MDA MB 435 cells (Fig. 4D; Fig. S5A). To test the dependence of the altered gene expression on MAPK signalling, exemplar genes were selected for analysis [seven upregulated genes (*HBEGF*, *CFS2*, *ENPP1*, *PLAU*, *PLAT*, *PTPRU* and *HSD11BA*) and one downregulated gene (*MXRA8*)], based on a sufficient number of sequence reads that would enable robust quantification by quantitative RT-PCR (qPCR) following 24 h treatment with vehicle DMSO or the allosteric MEK1/2 inhibitor Trametinib (Grimaldi et al., 2017; Roskoski, 2017). The expression of all seven upregulated genes identified by RNA-seq were expressed >100%, and *MXRA8* was <100%, in pore-selected Sel2 cells relative to Parent MDA MB 231 cells, and Trametinib significantly reversed these expression patterns (Fig. 4E). Similarly, the expression of six upregulated genes (*HBEGF*, *CFS2*, *PLAU*, *PLAT*, *PTPRU* and *HSD11BA*) and one downregulated gene (*MXRA8*) identified by RNA-seq were confirmed as being correspondingly altered by qPCR, and significantly reversed by Trametinib treatment in Sel1 MDA MB 435 cells (Fig. 4F).

**Fig. 5.**
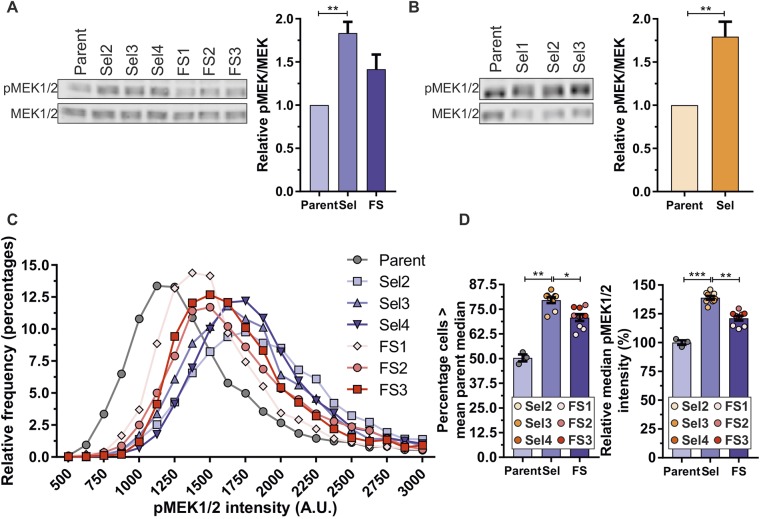
**Elevated active MEK in pore-selected cells.** (A) Left; western blots of phosphorylated active MEK1/2 (pMEK1/2) and total MEK1/2 (MEK1/2) for MDA MB 231 Parent, Sel2, Sel3, Sel4, FS1, FS2 and FS3 populations. Right, graph of the mean±s.e.m. ratio of pMEK1/2 to total MEK1/2 relative to Parent MDA MB 231 cells (set at 1). **P<0.01 (Kruskal–Wallis one-way ANOVA and Dunn's multiple comparisons test; Parent, *n*=3; Sel and FS, *n*=9 replicates). (B) Left, western blots of pMEK1/2 and MEK1/2 for Parent, Sel1, Sel2, and Sel3 populations. Right, Graph of the mean±s.e.m. ratio of pMEK1/2 to total MEK1/2 relative to Parent MDA MB 435 cells. **P<0.01 (Student's *t*-test; Parent, *n*=3; Sel, *n*=9 replicates). (C) Frequency distribution of MDA MB 231 Parent, three independent pore-selected (Sel2, Sel3 and Sel4) and three independent flow-sorted (FS1, FS2, FS3) of the mean pMEK1/2 single-cell fluorescence intensities at 250 arbitrary unit (A.U.) bins from three replicates. (D) Left graph, the mean±s.e.m. percentage of cells from the indicated Sel and FS populations with higher levels of pMEK1/2 the mean of Parent median determined from three replicate determinations. Right graph; the mean of the median pMEK1/2 fluorescence intensities of Parent cells from three replicate determinations was set 100%, and compared with the median pMEK1/2 for each replicate experiment for the cells indicated. Parent, *n*=3; Sel, *n*=9; FS *n*=9 replicates. *P<0.05, **P<0.01, ***P<0.001 (Kruskal–Wallis one-way ANOVA and Dunn's multiple comparisons test).

To assess MAPK activation, MDA MB 231 Parent, Sel and FS isolates were western blotted for active phosphorylated (pMEK1/2) and total MEK1 and MEK2 (MEK1/2) (Fig. 5A, left panel). By quantifying the relative intensities of near-infrared dye-conjugated secondary antibody staining, it was determined that Sel populations had significantly elevated ratio of active-to-total MEK1/2 indicating increased MAPK signalling (Fig. 5A, right panel; Fig. S5B). Similarly, the elevated ratio of active-to-total MEK1/2 was significantly higher in MDA MB 435 Sel populations relative to Parent cells (Fig. 5B; Fig. S5C). These results indicate that pore-selected Sel isolates have relatively higher levels of basal MAPK signalling. To determine whether pore-selection was likely to have enriched for cells from the MDA MB 231 Parent population for cells with pre-existing higher levels of MAPK signalling, the frequency distributions of pMEK1/2 staining intensities were plotted for >2800 single cells as determined by high-content imaging. Compared to the frequency distribution for Parent cells (Fig. 5C, grey line), there was a slight rightward shift for FS populations and an even greater rightward shift for Sel populations (Fig. 5C; FS cells, red lines; Sel cells, blue lines). When the median pMEK1/2 intensities for Parent cells from three independent experiments were used to determine the percentage of Sel and FS cells with higher pMEK1/2 levels than 50% of Parent cells [1245 arbitrary units (A.U.)], the Sel populations had significantly more cells with higher pMEK1/2 staining intensity than either Parent or FS cells (Fig. 5D, left panel). Similarly, when the median values for the three independent replicate experiments for the three Sel and FS populations were compared to Parent cells (1245±42 A.U.), Sel median pMEK1/2 levels were ∼39% higher (1729±60 A.U.) and FS median values were ∼21% higher (1508±76 A.U.). Importantly, the Sel median pMEK1/2 staining intensities were significantly higher than both Parent and FS populations (Fig. 5D, right panel). These observations support the conclusion that pore-selection enriched for cells with relatively higher MAPK signalling that pre-existed in the Parent population, similar to the enrichment for pre-existing smaller cells from the broader distribution of Parent cell sizes (Fig. 1C,E).

### MAPK is necessary and sufficient for changes in elasticity associated with invasion

**Fig. 6.**
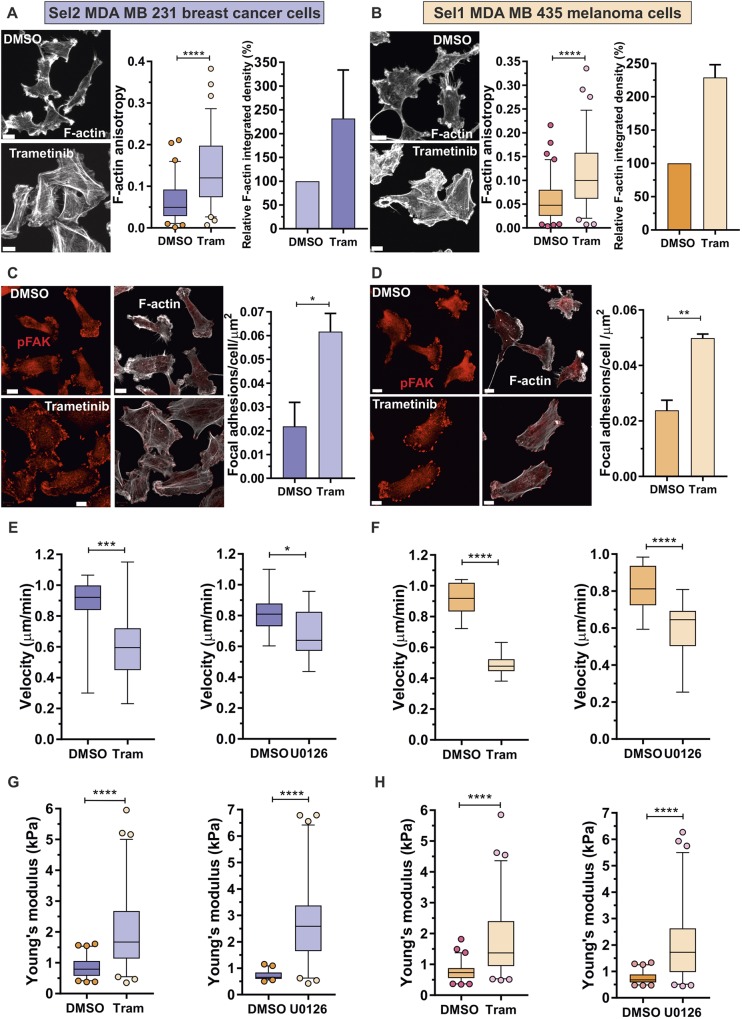
**F-actin anisotropy, focal adhesion density, motility and cell stiffness are influenced by MAPK activity.** (A) Left panels, Sel2 MDA MB 231 cells were treated with DMSO or 0.5 µM Trametinib for 24 h, then stained with phalloidin for F-actin. Scale bars: 10 µm. Middle panel, F-actin anisotropy was scored for DMSO (*n*=70) and Trametinib (Tram; *n*=71) treatments. ****P<0.0001 (Student's *t*-test). Right panel, relative mean±s.e.m. F-actin integrated density normalized to levels for cells treated with DMSO vehicle. DMSO, *n*=3; Trametinib, *n*=3 replicates. (B) Left panels, Sel1 MDA MB 435 cells were treated with DMSO or 0.5 µM Trametinib for 24 h, then stained with phalloidin for F-actin. Scale bars: 10 µm. Middle panel, F-actin anisotropy was scored for DMSO (*n*=85) and Trametinib (Tram; *n*=76) treatments. ****P<0.0001 (Student's *t*-test). Right panel, relative mean±s.e.m. F-actin integrated density normalized to levels for cells treated DMSO vehicle. DMSO, *n*=3; Trametinib, *n*=3 replicates. (C) Left panels, Sel2 MDA MB 231 cells were treated with DMSO or 0.5 µM Trametinib for 24 h, then stained for pFAK. Scale bars: 10 µm. Right panel, mean±s.e.m. pFAK-positive focal adhesion density was determined in three independent experiments for DMSO and Trametinib. *P<0.05 (Student's *t*-test). (D) Left panels, Sel1 MDA MB 435 cells were treated with DMSO or 0.5 µM Trametinib for 24 h, then stained for pFAK. Scale bars: 10 µm. Right panel; mean±s.e.m. pFAK-positive focal adhesion density was determined in three independent experiments with DMSO and Trametinib treatments. **P<0.01 (Student's *t*-test). (E) Mean velocity of Sel2 MDA MB 231 cells was determined over 22 h in three independent experiments with, left panel, DMSO vehicle or 0.5 µM Trametinib, or, right panel, DMSO or 10 µM U0126 treatments. *P<0.05, ***P<0.001 (Student's *t*-test). (F) Mean velocity of Sel1 MDA MB 435 cells was determined over 22 h in three independent experiments with, left panel, DMSO vehicle or 0.5 µM Trametinib, or, right panel, DMSO or 10 µM U0126 treatments. ****P<0.0001 (Student's *t*-test). (G) Young's modulus of Sel2 MDA MB 231 cells treated with, left panel, DMSO vehicle (*n*=62) or 0.5 µM Trametinib (*n*=67), or, right panel, DMSO vehicle (*n*=57) or 10 µM U0126 (*n*=65) for 24 h. ****P<0.0001 (Student's *t*-test). (H) Young's modulus of Sel1 MDA MB 435 cells treated with, left panel, DMSO vehicle (*n*=60) or 0.5 µM Trametinib (*n*=64), or, right panel, DMSO vehicle (*n*=63) or 10 µM U0126 (*n*=68) for 24 h. ****P<0.0001 (Student's *t*-test). For all box plots, the box represents the 25–75th percentiles, and the median is indicated. The whiskers show the 5–95th percentiles, and outliers are indicated.

**Fig. 7.**
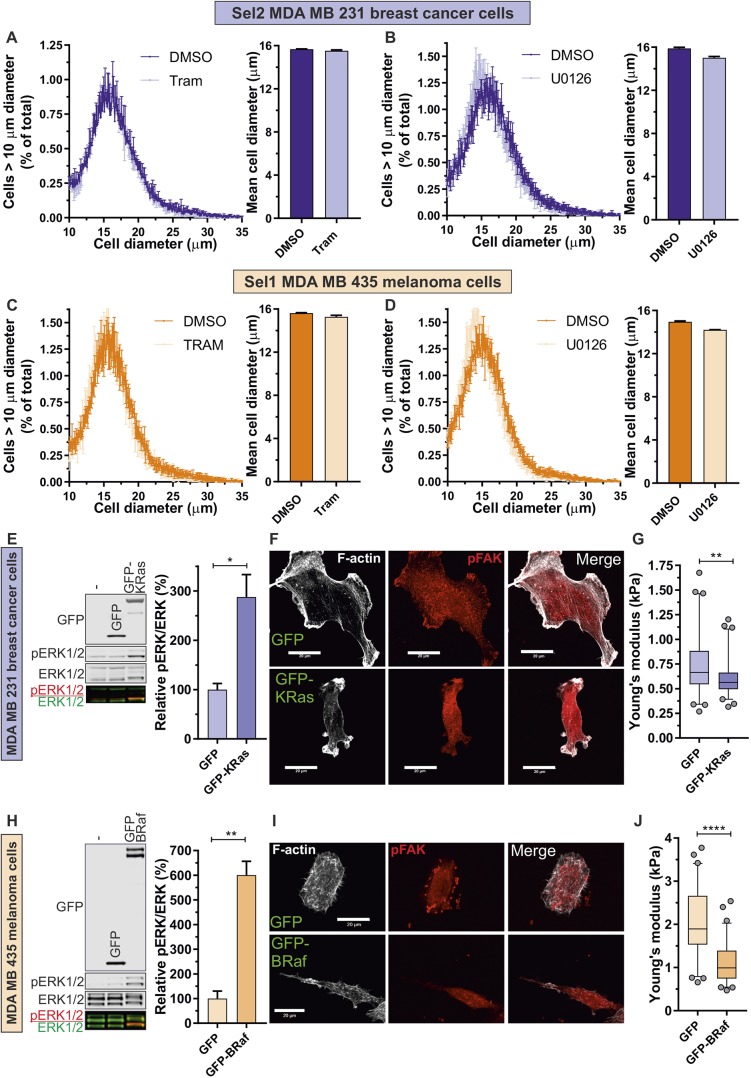
**Although MAPK inhibition does not alter cell size, increased MAPK signalling alters actin organization and reduces cell stiffness.** (A) Left, mean±s.d. cell diameter profiles for Sel2 MDA MB 231 cells treated with DMSO or 0.5 µM Trametinib for 24 h. Right, mean±s.e.m. cell profiles from DMSO- or Trametinib-treated cells from three independent determinations. (B) Left, mean±s.d. cell diameter profiles for Sel2 MDA MB 231 cells treated with DMSO or 10 µM U0126 for 24 h. Right, mean±s.e.m. cell profiles from DMSO or U0126 treated cells from three independent determinations. (C) Left, mean±s.d. cell diameter profiles for Sel1 MDA MB 435 cells treated with DMSO or 0.5 µM Trametinib for 24 h. Right, mean±s.e.m. cell profiles from DMSO- or Trametinib-treated cells from three independent determinations. (D) Left, mean±s.d. cell diameter profiles for Sel1 MDA MB 435 cells treated with DMSO or 10 µM U1026 for 24 h. Right, mean±s.e.m. cell profiles from DMSO- or U0126-treated cells from three independent determinations. Data for frequency distributions in panels A–D were plotted at 0.1 μm bins. (E) Parent MDA MB 231 cells were transfected with plasmids encoding GFP or GFP–KRas G12D, and enriched by flow sorting. Left panel, western blotting for GFP, pERK1/2 (red), total ERK1/2 (green). Right panel; GFP–KRas G12D significantly increased MAPK activation (ratio of pERK to total ERK). Results are mean±s.e.m. from three independent experiments. *P<0.05 (Student's *t*-test). (F) Parent MDA MB 231 cells transfected with plasmids encoding GFP or GFP–KRas G12D were fixed and stained with phalloidin to reveal F-actin structures (left) or pFAK to mark focal adhesions (middle). Scale bars: 20 µm. (G) Young's modulus of cells expressing GFP (*n*=60) or GFP-KRas G12D (*n*=61). **P<0.01 (Student's *t*-test). (H) Parent MDA MB 435 cells were transfected with plasmids encoding GFP or GFP–BRaf V600E, and enriched by flow sorting. Left panel, western blotting for GFP, active pERK1/2 (red) and total ERK1/2, (green). Right panel; GFP-BRaf V660E significantly increased MAPK activation (ratio of pERK to total ERK). Results are mean±s.e.m. from three independent experiments. **P<0.01 (Student's *t*-test). (I) Parent MDA MB 435 cells transfected with plasmids encoding GFP or GFP–BRaf V600E were fixed and stained with phalloidin to reveal F-actin structures (left) or pFAK to mark focal adhesions (middle). Scale bars: 20 µm. (J) Young's modulus of GFP (*n*=61) or GFP-BRaf V600E (*n*=64)-expressing cells. ****P<0.0001 (Student's *t*-test). For all box plots, the box represents the 25–75th percentiles, and the median is indicated. The whiskers show the 5–95th percentiles, and outliers are indicated.

The influence of MAPK signalling on F-actin cytoskeleton structures, focal adhesions and cell elasticity was examined by treating pore-selected cells with the MEK inhibitors Trametinib (Roskoski, 2017) or U0126 (Favata et al., 1998). F-actin structures were observably more extensive in pore-selected Sel2 MDA MB 231 cells following 0.5 µM Trametinib treatment (Fig. 6A, left panel), which was reflected by significantly increased F-actin anisotropy (Fig. 6A, middle panel) and greater F-actin staining (Fig. 6A, right panel) as determined from images obtained by TIRF microscopy (Fig. S6A). Similar observations were made following Trametinib treatment of pore-selected Sel1 MDA MB 435 cells (Fig. 6B, left panel; Fig. S6B), with increased F-actin anisotropy (Fig. 6B, middle panel) and greater F-actin staining (Fig. 6B, right panel). The MEK inhibitor U0126 (10 µM) also resulted in changes in actin cytoskeleton changes in both Sel2 MDA MB 231 cells (Fig. S6C) and Sel1 MDA MB 435 cells (Fig. S6D), which resulted in increased F-actin filament anisotropy and greater levels of F-actin staining in both cell types (Fig. S6E,F). Treatment with 0.5 µM Trametinib also significantly increased the pFAK-positive focal adhesion density in Sel2 MDA MB 231 (Fig. 6C; Fig. S6G) and Sel1 MDA MB 435 cells (Fig. 6D; Fig. S6H). Similar changes in focal adhesion density were induced in both cell types by treatment with 10 µM U0126 (Fig. S6I,J). The random single-cell velocities of Sel2 MDA MB 231 cells were significantly reduced by 0.5 µM Trametinib and 10 µM U0126 (Fig. 6E; Fig. S7A), as were the velocities of Sel1 MDA MB 435 cells (Fig. 6F; Fig. S7B). In parallel, the accumulated distances travelled by Sel2 MDA MB 231 (Fig. S7C) and Sel1 MDA MB 435 cells (Fig. S7D) were reduced by both Trametinib and U0126. The changes in F-actin organization and focal adhesions induced by 0.5 µM Trametinib and 10 µM U0126 were accompanied by significantly increased cell stiffness in Sel2 MDA MB 231 breast cancer cells (Fig. 6G) and Sel1 MDA MB 435 melanoma cells (Fig. 6H). However, the diameters of Sel2 MDA MB 231 cells were not increased by 0.5 µM Trametinib (Fig. 7A) or 10 µM U0126 (Fig. 7B), with similar observations of a lack of effect on MDA MB 435 cells treated with Trametinib (Fig. 7C) or U0126 (Fig. 7D). These results indicate that the majority of the phenotypes enriched by passage through 3 µm diameter pores that are associated with elevated MAPK activity (i.e. changes in gene expression, actin cytoskeleton rearrangements, decreases in focal adhesion density, increased motility and cell softening) could be reversed by inhibition of MAPK signalling. The exception was the selection for small diameter cells, which appears to be an independent variable that likely also contributes to passage through small physical constrictions.

Given that pore-selected cells showed evidence of increased MAPK signalling (Fig. 5) and MEK inhibitor-induced cell stiffening (Fig. 6G,H), one question was whether increased MAPK signalling would be sufficient to decrease Parent cell stiffness. Transfection and fluorescence-based sorting of Parent MDA MB 231 cells expressing GFP or activated GFP–KRas G12D (Fig. 7E, left panel) revealed significantly increased phosphorylated ERK1/2 (pERK1/2) (Fig. 7E, left and right panels; Fig. S5D), which was accompanied by reduced F-actin fibres, focal adhesion density (Fig. 7F) and significantly decreased cell stiffness (Fig. 7G). Similarly, transfection and sorting of Parent MDA MB 435 cells with GFP or activated GFP–BRaf V600E (Hernandez et al., 2016) (Fig. 7H, left panel) resulted in significantly increased pERK1/2 (Fig. 7H, left and right panels; Fig. S5E), which was accompanied by reduced F-actin fibres, focal adhesion density (Fig. 7I) and a significantly decreased cells stiffness (Fig. 7J). The greater magnitude of ERK1/2 activation induced by GFP–BRaf V600E expression in MDA MB 435 cells (Fig. 7H) relative to the GFP-KRas G12D expression in MDA MB 231 cells (Fig. 7E) was associated with a comparably larger decrease in cell stiffness (Fig. 7G,J), consistent with a direct link between MAPK activation and regulation of cell mechanical properties. These findings reveal that the Ras/RAF-regulated MAPK pathway has a profound role in the regulation of cancer cell biomechanics.

## DISCUSSION

From a biomechanical perspective, cells have viscoelastic properties that can deform if force is applied, and will recover their shape when force is removed (Kuznetsova et al., 2007). Changes in cellular mechanical properties have been observed in cancer, with tumour cells being softer than normal cells, and highly metastatic cells being more easily deformed than less metastatic cancer cells (Cross et al., 2007; Faria et al., 2008; Hayashi and Iwata, 2015; Li et al., 2008). These changes in mechanical properties have been proposed to play important roles in several of the steps in the metastatic process by increasing morphological plasticity to enable cells to pass through physical constrictions (Cross et al., 2007, 2008).

The actin cytoskeleton plays important roles in defining cell shape, driving migration and invasion, and is also a major factor affecting cell mechanical properties (Li et al., 2008; Palmieri et al., 2015). The main determinant of cell elasticity is the cortical cytoskeleton, the dense actin–myosin meshwork that lies directly beneath the plasma membrane (Bray and White, 1988; Salbreux et al., 2012). In addition to the density and length of actin-myosin filaments, the organization of cytoskeleton structures determines cell elasticity. Cells with well-aligned anisotropic actin–myosin fibres are stiffer, while cells with disorganized isotropic cytoskeleton structures are softer (Gupta et al., 2015). In this study, RNA sequencing identified increased KRas/MAPK signalling as a defining feature of pore-selected cells. Given that MDA MB 231 breast cancer cells express activated G13D KRas and moderately activated G464V BRaf (Davies et al., 2002), while MDA MB 435 melanoma cells express oncogenic V600E BRaf, increased KRas signal output was not the result of selection for cells with *de novo* Ras/MAPK pathway mutations. Instead, pore-selection enriched for cells with relatively higher Ras/MAPK signal output from the dispersed distribution of activation levels that would be present in the starting population. One question arising is whether selection for elevated Ras/MAPK signal output is dependent on the presence of activating Ras/MAPK mutations, or whether cells transformed by other oncogenes would also be enriched for elevated Ras/MAPK signalling to reduce cell stiffness when subjected to narrow pore-selection. An additional question is if acute Ras/MAPK activation during the migration of non-transformed cells, such as following ligand stimulation, would be sufficient to transiently reduce cell stiffness to enable migration through confined environments, as would occur, for example, during leukocyte extravasation, or whether sustained Ras/MAPK signal output is necessary to drive long-term adaptions, possibly mediated by transcriptional responses that alter biomechanical properties.

Interestingly, we observed that pore-selected MDA MB 435 melanoma cells were proportionally more invasive through 3 µm diameter pores (Fig. 1F) and fibroblast-conditioned collagen (Fig. 1J) than their Parent cells when compared to the lesser increase in invasiveness of pore-selected MDA MB 231 cells relative to their Parent cells (Fig. 1D,I). However, the absolute values for F-actin anisotropy (Fig. 3C,E), focal adhesion density (Fig. 3I,K) and elasticity (Fig. 3J,L) were not greatly different between the MDA MB 231 and MDA MB 435 pore-selected populations, nor were the relative fold-change differences in F-actin levels (Fig. 3D,F) or MEK phosphorylation (Fig. 5A,B). It is important to note that the MDA MB 231-luc-D3H2LN clone used in this study was previously selected *in vivo* for their ability to spontaneously disseminate to lymph nodes from their mammary fat pad site of injection (Jenkins et al., 2005). It is possible that the *in vivo* selection had already enriched for some properties that enabled efficient passage through narrow pores, such that there was a lower potential for large increases in invasive behaviour compared to what was possible for the MDA MB 435 cells, which had not been previously selected. One such property is the velocity of cell migration, which was approximately three times higher in Parent MDA MB 231 cells than in Parent MDA MB 435 cells (Fig. 1G,H). In addition, the greater number and magnitude of significantly changed mRNA transcripts in pore-selected MDA MB 435 cells relative to their Parent cells than for pore-selected MDA MB 231 cells compared to their Parent cells (Fig. 4A) suggests that the melanoma cell line may have greater potential for increased transcriptional responses, that collectively contribute to their proportionally larger increase in movement through narrow constraints.

The pore-selected cells were smaller in volume and two dimensional area than the parent cells for both MDA MB 231 and MDA MB 435 cells (Fig. 1C,E). By specifically selecting for small diameter cells by flow sorting, additional independent populations of small size MDA MB 231 cells were isolated, indicating that these small size cells exist in the parental population independent of the pore-selection. Indeed, the frequency distribution plots in Fig. 1C,E support the conclusion that the small size pore-selected and flow-sorted cells were selected from cells already present in the parental populations.

The nucleus is the largest and stiffest organelle, and previous studies have concluded that reduced nuclear stiffness is a major determinant of confined migration (McGregor et al., 2016). Pore-selection led to enrichment for small cell and nuclear size. However, analysis of small cells isolated by flow-sorting revealed that nuclear size was linked to cell size rather than the ability to undergo confined migration. Small nuclei in both pore-selected and flow-sorted isolates had fewer chromosomes, which were more compacted, and were stiffer than the larger nuclei in Parent cells. Given the strong link between cell and nucleus size (Edens et al., 2013), pore-selection likely co-selected both properties in Sel populations. Since equally small flow-sorted MDA MB 231 cells were not better than Parent cells at passing through 3 µm pores, small cell and nuclear size on their own are not sufficient to increase migration through pores, thereby reinforcing the importance of cytoskeleton-mediated elasticity and, by extension, the role of MAPK signalling in regulating cell biomechanical properties. These findings indicate that metastatic cancer cells benefit from MAPK signalling by decreasing cell stiffness to enable movement through confined environments, in addition to the significant role that MAPK signalling plays in the regulation of proliferation.

## MATERIALS AND METHODS

### Cell culture

MDA MB 231-luc-D3H2LN and MDA MB 435 cell lines were grown in HyClone MEM/EBSS medium (GE Healthcare Life Sciences, 11541871), supplemented with 10% fetal bovine serum (FBS) (Gibco, 10270), 2 mM L-glutamine (Gibco, 25030-032), 10 U/ml penicillin and 10 µg/ml streptomycin (Gibco, 15140-122), 1% MEM/NEAA (Thermo Fisher Scientific, 11140035), 1% sodium pyruvate (Thermo Fisher Scientific, 11360070). Cell identities were validated by the Cancer Research UK Beatson Institute Molecular Services using the GenePrint 10 system STR multiplex assay (Promega), which amplifies nine tetranucleotide repeat loci and the amelogenin gender-determining marker. Human fibroblasts were kindly provided by Max Nobis (Cancer Research UK Beatson Institute), and were grown in DMEM (Gibco, 21969-035) supplemented with 10% FBS, 2 mM L-glutamine, 10 U/ml penicillin and 10 µg/ml streptomycin. All cell lines were routinely tested for mycoplasma by the Cancer Research UK Beatson Institute Molecular Services.

Independent MDA MB 231 or MDA MB 435 pore-selected (Sel) populations were established by seeding 10^6^ cells in 10 ml serum-free medium on 3 μm pore membranes in 7.5 cm cell culture inserts (Corning, Thermo Fisher Scientific, 3420). Inserts were placed in 10 cm dishes containing 10 ml serum-containing medium, and were left for 5 days in standard tissue culture conditions to allow cells to migrate through the pores. The inserts were removed, the medium changed and plates were placed back in the incubator to expand the selected cell populations. The selection process was repeated twice more as described above.

Independent small diameter MDA MB 231 flow cytometry-sorted (flow-sorted; FS) populations were obtained by gating with low forward scatter (FSC) and side scatter (SSC) parameters using a FACSAria Fusion sorter (BD Biosciences, Oxford, UK). FS cells were grown using standard tissue culture conditions to expand the isolated sorted cell populations, followed by two additional rounds of sorting as described above.

Treatment of MDA MB 231 and MDA MB 435 cells with Trametinib (Medchem Express, HY-10999) or U0126 (Cell Signalling Technology, 9903S) inhibitors were performed as follows. Cells were seeded in culture dishes and grown overnight. Following incubation, 0.5 µM Trametinib or 10 µM U0126 was added and incubated for 24 h. After incubation, cells were processed depending on the experimental requirements. Both drugs were dissolved in DMSO (Sigma, D2650) to 10 mM stock concentrations. Treatment of MDA MB 231 cells with cytochalasin D (Sigma, 8273) was performed as follows. Cells were seeded in culture dishes and grown overnight. On the next day, 0.5 µM cytochalasin D was added and incubated for 2 h. After incubation, cells were processed depending on the experimental requirements. Cytochalasin D was dissolved in DMSO to a 10 mM stock concentration.

### Transwell cell migration assay

1.2×10^5^ cells for 3-µm-pore membrane inserts (Corning, 3415) and 6×10^4^ cells for 8-µm-pore membrane inserts (Corning, 4322) were re-suspended in 300 µl of serum-free medium. Cell suspensions were added into inserts and placed in wells of 24-well plates containing 750 µl of serum-containing medium. Plates were left for 3 days in standard culture conditions to allow cells to migrate through membranes and attach to the lower chamber compartment. The number of migrated cells at the bottom was quantified using a sulforhodamine B (SRB) (Sigma, 230162) colorimetric assay. After removing inserts, 10% (w/v) trichloroacetic acid (TCA) (Sigma, T6399) was added to each well to reach a final concentration of 3.3%. The cells were fixed at 4°C for 30 min. Cells were washed three times with water, air-dried and 300 µl of 0.04% of SRB was added to each well and incubated for 30 min at room temperature. After incubation, SRB solution was removed, wells were washed three times with 1% (v/v) acetic acid and air dried. Then, 250 µl of 10 mM Tris-HCl (pH 10.5) was added into each well and plates were placed on a shaker for 15 min. After incubation, 200 µl from each well was transferred to wells of 96-well plates and absorbance was measured on a spectrophotometer at 490 nm. For each independent experiment, three inserts were used per condition.

### Cell and nuclear volume

Cell volumes (*V*) were calculated using the formula *V*=¾π*r*^3^. Mean diameter measurements were determined using a CASY^®^ Cell Counter. For each independent mean diameter determination, >5000 cells were counted per replicate determination

Nuclei volumes were calculated using Volocity Software. *Z*-stack images of DAPI-stained nuclei were taken on a Zeiss 710 confocal microscope. The obtained *z*-stacks were analysed for 3D volumes using Volocity Software (Quorum Technologies), with 32–74 nuclei measured per condition for each independent experiment.

### Morphological cell properties

To determine cell morphological features including cell and nuclear areas, an Operetta high-content imaging system was used. Prior to cell imaging, cells were seeded in black 96-well plates at 2×10^4^ cells per well and grown overnight. After incubation, cells were fixed with 4% (w/v) paraformaldehyde (PFA; EMS, 15710) in PBS for 15 min and permeabilized with 0.5% (v/v) Triton X-100 (Thermo Fisher Scientific, 28314) for 5 min at room temperature. Cells were incubated with 0.15 µg/ml DAPI (Sigma, D9542) for 20 min at room temperature and subsequently with 50 µl of 1:5000 dilution of Cellomics^®^ Whole Cell Stain (WCS; Thermo Fisher Scientific, 8303401) for 30 min. Plates were imaged on an Operetta high-content imaging system and data was analysed using a Columbus™ image data storage and analysis System (PerkinElmer).

### Cytogenetic analysis of chromosome numbers

Metaphase spreads were produced by standard methods (Moralli et al., 2011). Briefly, subconfluent cells were incubated in Colcemid (Thermo Fisher Scientific) 30 ng/ml for 3 h, then cells were detached and swelled in Buffered Hypotonic Solution (Genial Genetics) for 30 min. Following two fixations in cold Carnoy's fixative (methanol:acetic acid at 3:1), cell suspensions were dropped on slides. Cells were mounted in Vectashield containing DAPI (Oncor) and analysed with an Olympus BX60 microscope for epifluorescence equipped with a Sensys CCD camera (Photometrics). Images were collected and analysed using the Genus Cytovision software (Leica). Chromosome numbers were counted in a minimum of 20 cells per line. The analysis was repeated for a total of three independent replicates.

### 3D collagen matrix invasion assay

Collagen matrix invasion assay was performed as described previously (Rath et al., 2017). MEM/EBSS complete medium containing 10 µM U0126 or DMSO was added to the dishes as indicated. Medium was changed every other day. H&E-stained sections were scanned using Leica Biosystems software (Leica). The invading distance of the cells was measured using ImageJ software (NIH Image/ImageJ). For each independent experiment, three or four collagen matrices were used per condition.

### RNA-Seq and qPCR

RNA-Seq and qPCR was performed as described previously (Rudzka et al., 2017).

### FLIM-FRET

H2B–GFP (from Addgene vector 11680) and H2B–mCherry (from Addgene vector 20972), were introduced into MDA MB 231 cells by electroporation using an Amaxa Cell Line Nucleofactor^®^ Kit V (Lonza, VCA-1003). 2 µg of DNA was introduced into cells according to manufacturer's instructions. Transfected cells were seeded on 35 mm glass-bottom dishes and left for 72 h in standard tissue culture conditions. After incubation, cells were imaged using FLIM microscopy. For 2-deoxyglucose (2-DG) and sodium azide treatment, cells were treated with 50 mM 2-deoxyglucose (Sigma, D8375) and 10 mM sodium azide (Sigma, S8032) 30 min before acquiring images. Control cells were treated with H_2_O.

FLIM images were taken on a Nikon TE 2000 inverted microscope fitted with a Lambert Instruments FLIM Attachment (LIFA) and a Yokogawa CSU22 spinning disk unit with 100× objective. The microscope was equipped with an incubation chamber suitable to maintain live cells and optics at constant temperature. The LIFA system was equipped with an Omicron 50 mW 445 nm laser for CFP lifetimes and a 60 mW 488 nm laser for GFP lifetimes. The experiment is based on the frequency domain method for fluorescence lifetime imaging and allows the rapid acquisition and generation of lifetime images. Fluorescence lifetimes were measured using LI-FLIM software (Lambert Instruments).

### Plasma membrane fluidity determination using FRAP

2×10^5^ cells were seeded on 35 mm glass-bottom dishes and incubated overnight. After incubation, cells were washed twice with HBSS medium followed by staining with NBD-sphingomyelin (Insight Biotechnology, 60031) at 4 µM for 10 min in room temperature protected from light. Cells were rinsed and observed in HBSS medium (Thermo Fisher Scientific, 14065-049). Evaluation of membrane fluidity was obtained by measuring lateral diffusion of NBD-sphingomyelin. All experiments were performed on a Zeiss 880 confocal microscope with a 60× oil immersion objective was used. For photobleaching, a 488 nm laser was used, set to 100% laser power. Images were set to 510×200 pixels and speed 12 for sufficient speed scanning. The zoom factor was set to 2 to avoid bleaching during acquisition. For bleaching a region of interest (ROI1), a circle of 24 pixels was defined on the apical cell body membrane. In order to perform quantification, two other regions of interests were drawn around the cell (ROI2) and a random non-fluorescent region, also referred to as background (ROI3). The mobile fraction and 50% fluorescent recovery (*t*_1/2_) were quantified using Easy FRAP software (omicX). The fluorescence intensity was normalized using a full-scale normalization method. In order to extract quantitative information from the curves, double-term fitting exponential equations were used.

### Force indentation acquisition by AFM

Mechanical properties of individual cells were measured using an atomic force microscope NanoWizard II (JPK Instruments) mounted onto an inverted optical microscope (Zeiss Axio Observe) with a cell heater attachment. Force indentation measurements were carried out using in-house-prepared AFM colloidal probes (4.74 µm spherical silica bead cantilevers, with a typical spring constant of ∼0.02 N/m) as described previously (McPhee et al., 2010). Calibration measurements were performed before every experiment to determine the spring constant for each cantilever as described previously (Hutter and Bechhoefer, 1993). Cells were cultured overnight prior to force indentation measurements. During the experiment, cells were kept at 37°C and in 1% HEPES buffered full medium to maintain pH levels. Indentations were performed at a loading force of 3 nN and a constant speed of 2.5 µm/s. The Young's modulus was derived by fitting a force–distance curve with the Hertzian spherical model (Lin et al., 2007). Five points on the central nucleus area of a cell were measured, and the highest Young's modulus value was used for the cell. Comparative studies between different populations were conducted using the same AFM probe and under the same conditions.

For transfections with GFP, GFP–KRas G12D (gift from Shehab Ismail, CRUK Beatson Institute) and GFP–BRaf V600E (gift of Catrin Pritchard, University of Leicester, UK) (Hernandez et al., 2016), cells were plated at 5×10^4^ per dish in 2 ml medium, and then transfected with FuGENE (Promega) according to manufacturer's instructions. After 5 h, medium was changed and cells left overnight. Elasticity measurements were as described above.

For the nuclei elasticity measurements, nuclei were isolated using the Nuclei EZ Prep Nuclei Isolation kit (Sigma, NUC101) according to manufacturer's instructions. Isolated nuclei were seeded in 3 cm petri dishes in the lysis buffer provided with the kit. Once nuclei had attached onto the dish, AFM was performed as described above. One measurement was taken per nucleus.

### Immunofluorescence

Immunofluorescence images were taken on Zeiss 710 upright confocal microscope or Zeiss 880 confocal using 63× oil immersion objectives. Primary pFAK (pY397) (BD Bioscience, 611722) antibodies were routinely used at 1:200 dilutions. Alexa Fluor 488-conjugated phalloidin (Thermo Fisher Scientific, A12379), and Alexa Fluor 594 conjugated (Thermo Fisher Scientific, A11032) secondary antibodies were used at 1:1000 dilution. Fluorescently labelled cells were mounted with ProLong Diamond including DAPI (Thermo Fisher Scientific, P36961).

Cells were fixed with 4% (w/v) PFA in PBS at room temperature for 15 min. Cells were permeabilized with 0.5% (v/v) Triton X-100 in PBS for 5 min at room temperature and non-specific binding was blocked with 1% BSA in PBS for 30 min at room temperature. Primary antibodies were incubated for 2 h at room temperature while secondary antibodies were incubated for 1 h at room temperature.

### TIRF microscopy and F-actin anisotropy

TIRF images were taken on a Nikon Eclipse TE2000-U microscope. A 100×1.45 NA Plan Apochromat TIRF oil objective (Nikon) was used. The microscope was equipped with a Photometrics Evolve 512 camera, which allowed illumination of GFP and RFP at 473 nm and 561 nm, respectively. Red and green fluorescence signals were separated using a DualView DV2 emission splitter. MetaMorph software was used to control camera shutters and light source. Samples for imaging were prepared as described for immunofluorescence. In order to quantify F-actin anisotropy, the ImageJ plugin FibrilTool was used (Boudaoud et al., 2014).

### Scanning electron microscopy

Isolated nuclei were seeded on 10 mm coverslips placed in 24-well plates and cultured overnight. After incubation, they were fixed with 3% glutaraldehyde in 0.1 M phosphate buffer for 2 h at room temperature. After fixation, nuclei were washed three times with 0.1 M phosphate buffer for 10 min each. Scanning electron microscopy was performed as described previously (Wickman et al., 2013).

### Imaging cell projections into microporous membranes

Cells grown on membrane filters were washed in PBS before fixing in 1.5% glutaraldehyde/0.1 M sodium cacodylate buffer for 1 h at 4°C then washed three times for 5 min each in 0.1 M sodium cacodylate buffer containing 2% (w/v) sucrose. Samples were post-fixed in 1% osmium tetroxide/0.1 M sodium cacodylate buffer for 1 h, then washed three times for 10 min each time in distilled water followed by *en block* staining in 0.5% aqueous uranyl acetate for 1 h (in dark–light-sensitive stain) and washed again twice for 1 min with distilled water. Dehydration steps were through a graded ethanol series (30%, 50%, 70% and 90%) for 10 min each, then 100% ethanol four times for 5 min each time, followed with propylene oxide three times for 5 min each time, and then 1:1 propylene oxide:Araldite/Epon resin (TAAB 812) overnight. Samples were then put into fresh pure Epon/Araldite resin, embedded in flat-bed moulds and polymerized for 48 h at 60°C.

Light microscope semi-thin sections (350 nm) were cut using a LEICA Ultracut UCT (Leica Microsystems) and Diatome diamond Histo-knife (Diatome) at an angle of 6°. Sections floating on water were stretched with chloroform vapour, picked up using a wire loop placed onto a droplet of distilled water and dried on a glass slide, using a hotplate set at 80°C. Sections were stained for 15–30 s with 1% Toluidine Blue stain, washed with 1% Borax with fresh distilled water and left to dry before mounting with a glass coverslip using DPX.

### Transfection, cell sorting and immunoblotting

To transfect Parent MDA MB 231 or MDA MB 435 cells, they were initially plated at 1.5×10^6^ cells per 10 cm diameter plate. For each GFP expression plasmid, 10 μg DNA was diluted into 500 µl jetPRIME^®^ buffer (Polyplus, 114-07) and mixed by vortexing; then 20 μl jetPRIME^®^ was added, mixed by vortexing, and plates were incubated for 10 min at room temperature. The transfection mix was added drop-wise onto cells in each plate with serum-containing medium, and distributed evenly by gentle rocking. Medium was replaced 4 h later with complete medium, and then cells were incubated overnight. Cells in each conditioned were trypsinized and pooled at a final concentration of 10^6^ cells/ml in medium with 1% FBS, and sorted for green fluorescence using a BD FACSAria™ with a 100 µm nozzle at 20 psi.

Standard protocols were used for western blot analysis as described previoulsy (Rath et al., 2017). Whole-cell lysates were prepared in cell lysis buffer (1% SDS, 50 mM Tris-HCl pH 7.5 plus protease inhibitors), and protein concentrations were determined by performing a Bicinchoninic assay (Sigma, B9643). Primary antibodies were routinely used at 1:1000 dilutions. Primary antibodies used were against GFP (Abcam, ab6556), pERK (Cell Signaling Technology, 9106), ERK (Cell Signaling Technology, 9102). Secondary antibodies used were goat anti-rabbit IgG (H+L) highly cross-adsorbed antibody conjugated to Alexa Fluor 680 (Thermo Fisher Scientific; A21109), goat anti-mouse IgG (H+L) highly cross-adsorbed antibody conjugated to Alexa Fluor 680 (Thermo Fisher Scientific; A21058) and IR Dye 800CW- conjugated donkey anti-rabbit-IgG (LICOR; 925-32213) at 1:5000 dilutions, and were detected by infrared imaging (Li-Cor Odyssey CLx).

## Supplementary Material

Supplementary information
