# Supplementary material for: Migration through physical constraints is enabled by MAPK-induced cell softening via actin cytoskeleton re-organization
Source: J Cell Sci. 2019 May 31;132(11):jcs224071. doi: 10.1242/jcs.224071 (PMC6589089; doi:10.1242/jcs.224071)
Supplement: Supplementary information [file joces-132-224071-s1.pdf]

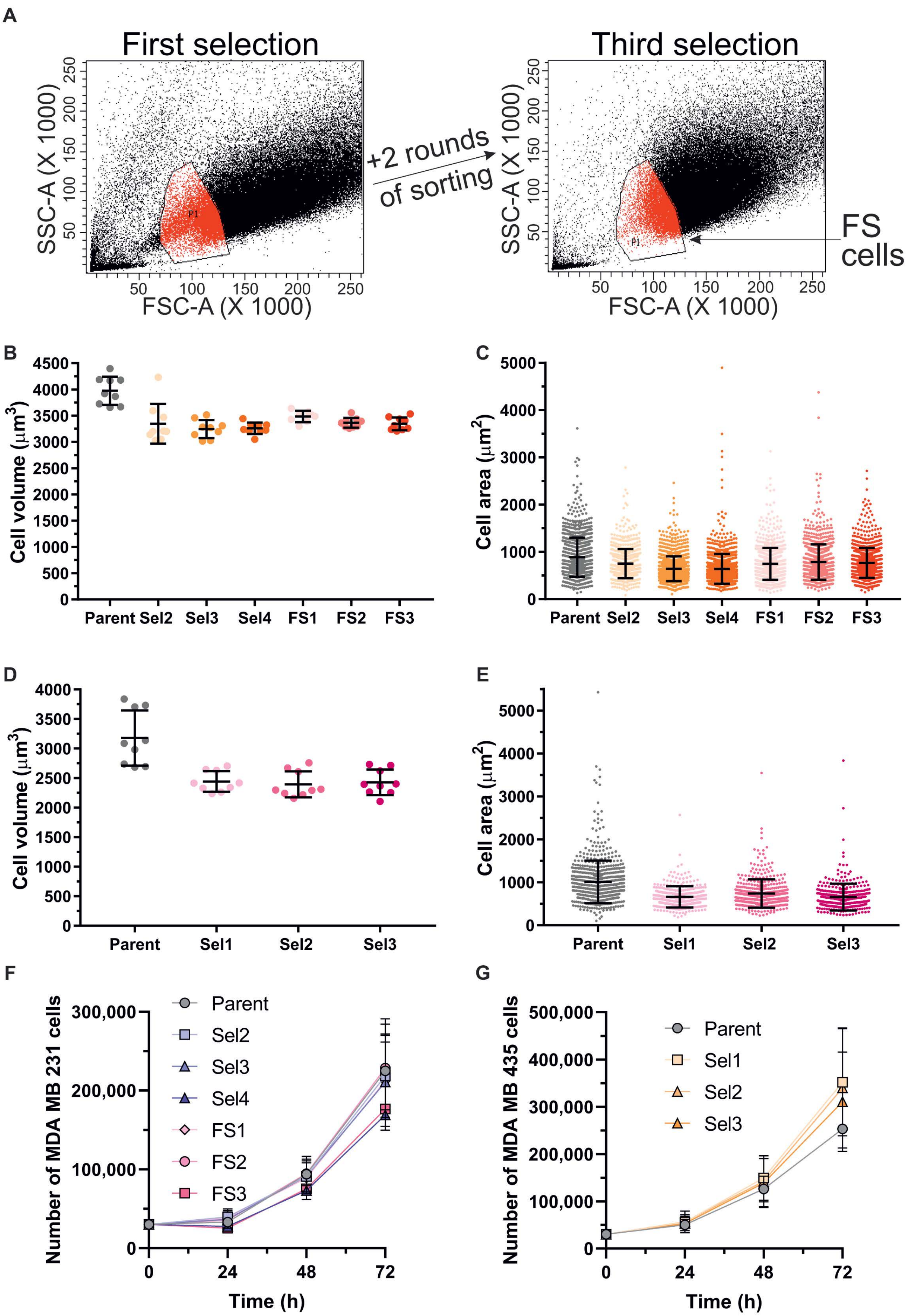

Journal of Cell Science • Supplementary information

Fig S1. (related to Fig 1) Flow sorting enrichment strategy and characterization of cell properties. A. Flow cytometry was used to enrich for small MDA MB 231 cells. Side scatter (SSC-A) and forward scatter (FSC-A) were used to gate for small cells, indicated in P1 region in red. After the first round of selection (left panel), two additional rounds were used to isolate three independent Flow Sorted (FS) populations (*e.g.* right panel). **B.** The volumes of cells in suspension were determined in independent replicates in a single experiment by measuring the diameters of > 5000 cells, in order to generate a single mean value in **Fig 1C**, *middle graph*. The mean volume determinations was determined for 9 experimental replicates of suspended MDA MB 231 cells, except for FS1 for which 8 replicates were assayed. Means ± SD. **C.** Example high content imaging experiment used to generate a single mean cell area in **Fig 1C**, *right graph*. The 2D area was determined for the following number of fixed MDA MB 231 cells: Parent (1120), Sel2 (724), Sel3 (2034), Sel4 (1237), FS1 (896), FS2 (966), FS3 (1034). Means ± SD. **D.** The volumes of cells in suspension were determined in independent replicates in a single experiment by measuring the diameters of > 5000 cells, in order to generate a single mean value in **Fig 1E**, *middle graph*. The mean volume determinations were determined for 9 experimental replicates of suspended MDA MB 435 cells. Means ± SD. **E.** Example high content imaging experiment was used to generate a single mean cell area in **Fig 1E**, *right graph*. The 2D area was determined for the following number of fixed MDA MB 435 cells: Parent (649), Sel1 (321), Sel2 (419), Sel3 (369). Means ± SD. **F.** Numbers of MDA MB 231 Parent, Sel2, Sel3, Sel4, FS1, FS2 or FS3 cells at indicated times after plating 30,000 cells. Triplicate determinations for all but Sel4 and FS3 which were done once. Means ± SD. **G.** Numbers of MDA MB 435 Parent, Sel1, Sel2 or Sel3 cells at indicated times after plating 30,000 cells in triplicate determinations. Means ± SD.

Journal of Cell Science • Supplementary information

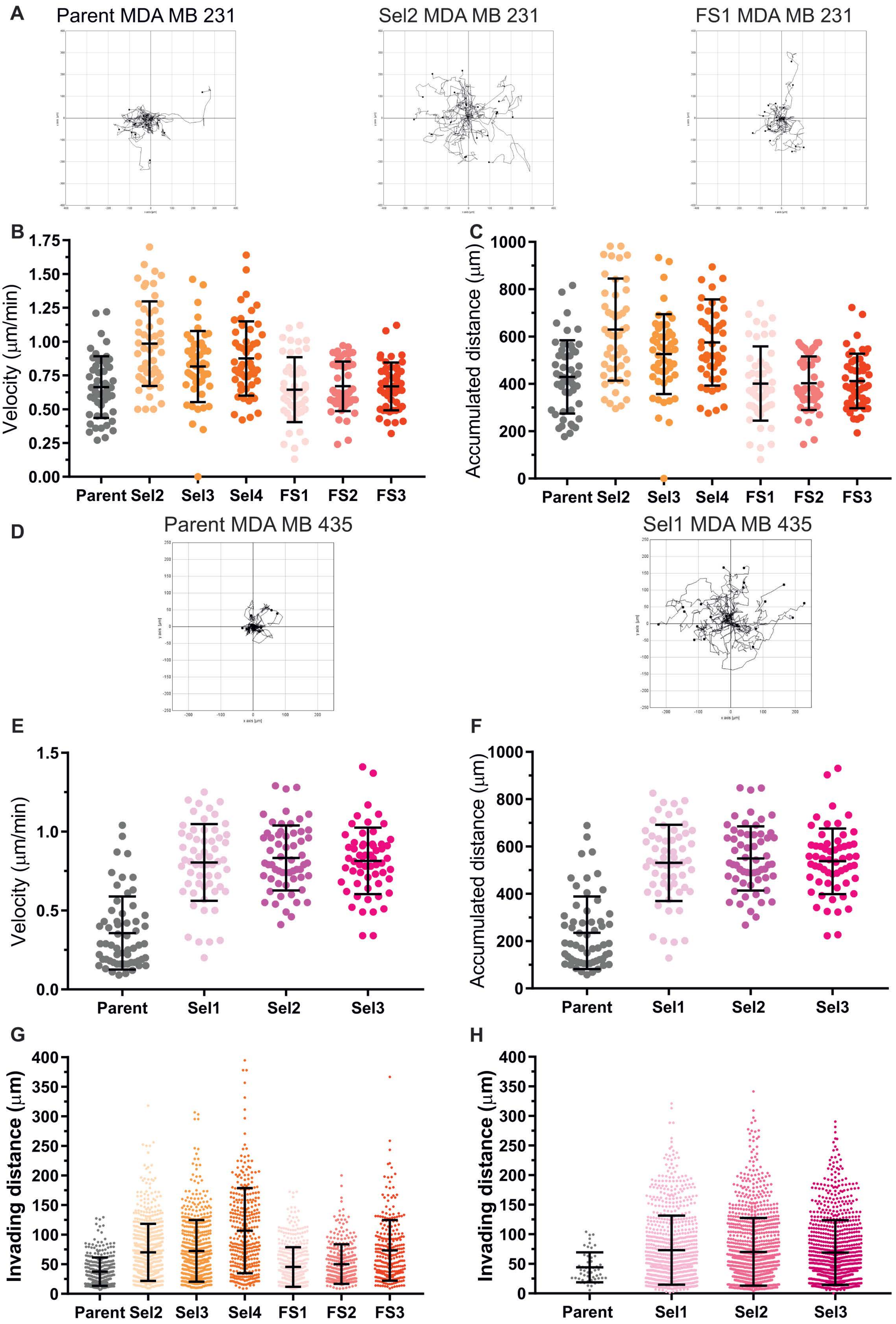
A
Parent MDA MB 231
Sel2 MDA MB 231
FS1 MDA MB 231
B
Velocity (µm/min)
Parent Sel2 Sel3 Sel4 FS1 FS2 FS3
C
Accumulated distance (µm)
Parent Sel2 Sel3 Sel4 FS1 FS2 FS3
D
Parent MDA MB 435
Sel1 MDA MB 435
E
Velocity (µm/min)
Parent Sel1 Sel2 Sel3
F
Accumulated distance (µm)
Parent Sel1 Sel2 Sel3
G
Invading distance (µm)
Parent Sel2 Sel3 Sel4 FS1 FS2 FS3
H
Invading distance (µm)
Parent Sel1 Sel2 Sel3

Fig S2. (related to Fig 1) Single cell migration and invasion distances by individual cells into fibroblast conditioned collagen. A. Example traces of randomly migrating MDA MB 231 Parent (*left panel*), Sel2 (*middle panel*) and FS1 (*right panel*) cells tracked over 22 hours. **B**. Example cell tracking experiment that was used to generate a single mean single cell velocity in **Fig 1G**, *left graph*. The random migration velocities were determined for the following number of live MDA MB 231 cells: Parent (49), Sel2 (51), Sel3 (49), Sel4 (50), FS1 (48), FS2 (48), FS3 (48). Means ± SD. **C.** Example cell tracking experiment that was used to generate a single mean accumulated distance for MDA MB 231 cells in **Fig 1G**, *right graph*. The accumulated distance values were extracted from the same cell tracking experiment as in **Fig S2B**. Means ± SD. **D.** Example traces of randomly migrating MDA MB 435 Parent and Sel1 cells tracked over 22 hours. **E**. Example cell tracking experiment that was used to generate a single mean single cell velocity in **Fig 1H**, *left graph*. The random migration velocities were determined for 59 live MDA MB 435 cells for each of Parent, Sel1, Sel2 and Sel2 populations. Means ± SD. **F.** Example cell tracking experiment that was used to generate a single mean accumulated distance for MDA MB 435 cells in **Fig 1H**, *right graph*. The accumulated distance values were extracted from the same cell tracking experiment as in **Fig S2E**. **G.** Example experiment in which the distances invaded by individual cells from the surface of a single 3D collagen matrix, which was used to generate a single mean value in **Fig 1I**. The invading distances were determined for the following number of MDA MB 231 cells: Parent (322), Sel2 (635), Sel3 (551), Sel4 (342), FS1 (451), FS2 (301), FS3 (283). Means ± SD. **H.** Example experiment in which the distances invaded by individual cells from the surface of a single 3D collagen matrix, which was used to generate a single mean value in **Fig 1J**. The invading distances were determined for the following number of MDA MB 435 cells: Parent (52), Sel1 (831), Sel2 (1111), Sel3 (910). Means ± SD.

Journal of Cell Science • Supplementary information

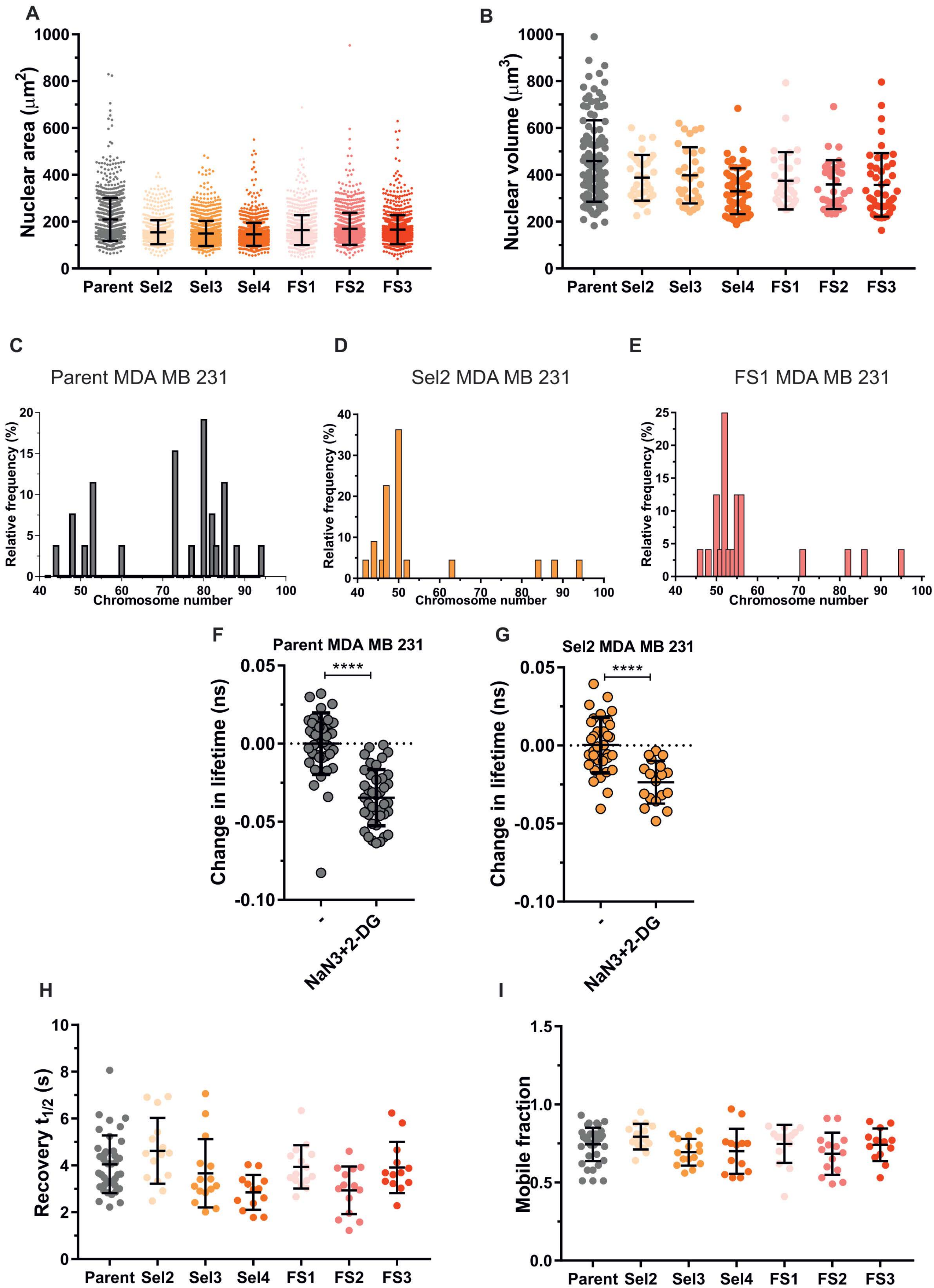
A
Nuclear area (μm²)
Parent Sel2 Sel3 Sel4 FS1 FS2 FS3
B
Nuclear volume (μm³)
Parent Sel2 Sel3 Sel4 FS1 FS2 FS3
C
Parent MDA MB 231
Relative frequency (%)
Chromosome number
D
Sel2 MDA MB 231
Relative frequency (%)
Chromosome number
E
FS1 MDA MB 231
Relative frequency (%)
Chromosome number
F
Parent MDA MB 231
Change in lifetime (ns)
****
-
NaN3+2-DG
G
Sel2 MDA MB 231
Change in lifetime (ns)
****
-
NaN3+2-DG
H
Recovery t1/2 (s)
Parent Sel2 Sel3 Sel4 FS1 FS2 FS3
I
Mobile fraction
Parent Sel2 Sel3 Sel4 FS1 FS2 FS3

Fig S3. (related to Fig 2) Nuclear properties of Parent and selected cells. A. Example high content imaging experiment used to generate a single mean nucleus area in **Fig 2A**, *left graph*. The nucleus area was determined for the following number of fixed MDA MB 231 cells: Parent (1120), Sel2 (724), Sel3 (2034), Sel4 (1237), FS1 (896), FS2 (966), FS3 (1034). Means ± SD. **B.** Individual nuclear volume data points acquired by confocal microscopy in **Fig 2A**, *right graph.* The nucleus volumes were determined for the following number of fixed MDA MB 231 cells. Parent n = 120; Sel2 (34), Sel3 (31), Sel4 (58), FS1 (32), FS2 (33) or FS3 (46). **C.** Example experiment in which the relative frequency of Parent MDA MB 231 cells (n = 26) with the indicated number chromosomes was used to generate a single mean chromosome number in **Fig 2B**. **D.** Example experiment in which the relative frequency of Sel2 MDA MB 231 cells (N = 22) with the indicated number chromosomes was used to generate a single mean chromosome number in **Fig 2B**. **E.** Example experiment in which the relative frequency of FS1 MDA MB 231 cells (n = 24) with the indicated number chromosomes was used to generate a single mean chromosome number in **Fig 2B**. **F.** Control experiment to validate the FRET-FLIM method for **Fig 2C** to compare chromatin compaction. For Parent MDA MB 231 cells, 41 untreated and 40 NaZ + 2-DG treated cells were assayed. Means ± SD. **G.** Control experiment to validate the FRET-FLIM method for **Fig 2C** to compare chromatin compaction. For Sel2 MDA MB 231 cells 33 untreated and 18 NaZ + 2-DG treated cells were assayed. Means ± SD. **H.** Individual $t_{1/2}$ recovery times determined following FRAP that were used in **Fig2F**, *middle graph*. The $t_{1/2}$ recovery times were determined for the following number of live MDA MB 231 cells: Parent (39), Sel2 (15). Sel3 (15), Sel4 (14), FS1 (15), FS2 (14), FS3 (13). **I.** Individual mobile fractions determined following FRAP determined that were used in **Fig2F**, *right graph*. The mobile fractions were determined for the following number of live MDA MB 231 cells: Parent (39), Sel2 (15). Sel3 (15), Sel4 (14), FS1 (15), FS2 (14), FS3 (13).

Journal of Cell Science • Supplementary information

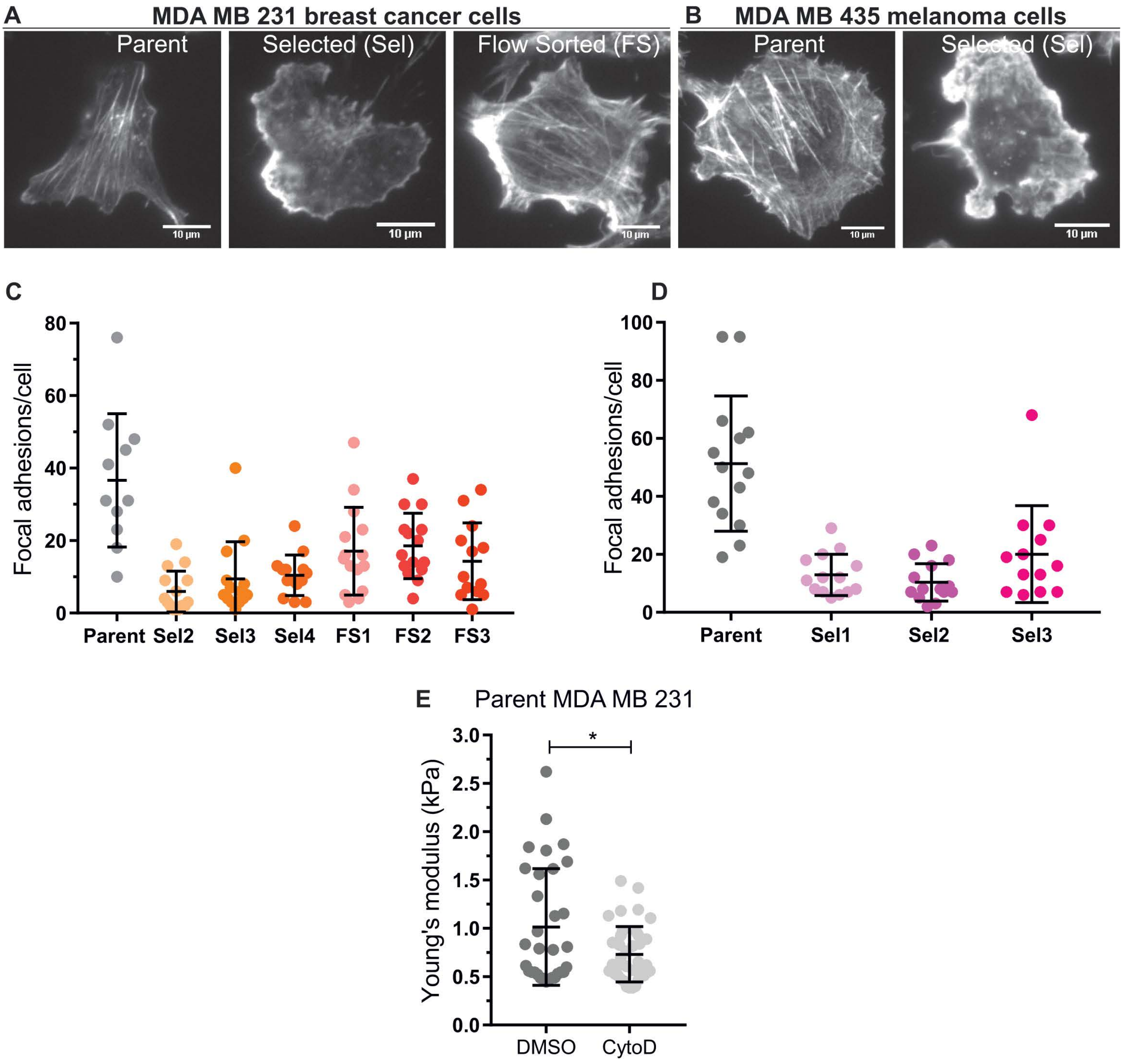

A
MDA MB 231 breast cancer cells
Parent
Selected (Sel)
Flow Sorted (FS)
10 µm
B
MDA MB 435 melanoma cells
Parent
Selected (Sel)
C
Focal adhesions/cell
Parent
Sel2
Sel3
Sel4
FS1
FS2
FS3
D
Focal adhesions/cell
Parent
Sel1
Sel2
Sel3
E
Parent MDA MB 231
Young's modulus (kPa)
*
DMSO
CytoD

Fig S4. (related to Fig 3) TIRF images of F-actin and focal adhesion numbers in Parent and Selected cell populations. A. Total internal reflection fluorescence (TIRF) microscopic images of representative Parent, Selected (Sel) and Flow-sorted (FS) MDA MB 231 and **B.** Parent and Selected (Sel) MDA MB 435 cells that had been fixed and stained for filamentous actin (F-actin) structures with fluorescently-labelled phalloidin. Images such as those presented were analysed with the ImageJ plugin FibrilTool [1] with corresponding results in **Figs 3C,D** *left panels*. **C.** Example imaging experiment that was used to generate a single mean focal adhesion density in **Fig 3I**. The focal adhesion numbers per cell were determined for the following number of fixed MDA MB 231 cells: Parent (11), Sel2 (15), Sel3 (14), Sel4 (14), FS1 (15), FS2 (15), FS3 (13). Means ± SD. **D.** Example imaging experiment that was used to generate a single mean focal adhesion density in **Fig 3K**. The focal adhesion numbers per cell were determined for the following number of fixed MDA MB 435 cells: Parent (14), Sel1 (14), Sel2 (14), Sel3 (13). Means ± SD. **E.** The elasticity (Young's modulus) of Parent MDA MB 231 cells was determined following treatment with vehicle DMSO (n = 32) or 0.5 µM Cytochalasin D (n =39) for 2 hours. Student's t-test (* = $p<0.05$). Means ± SD.

Journal of Cell Science • Supplementary information

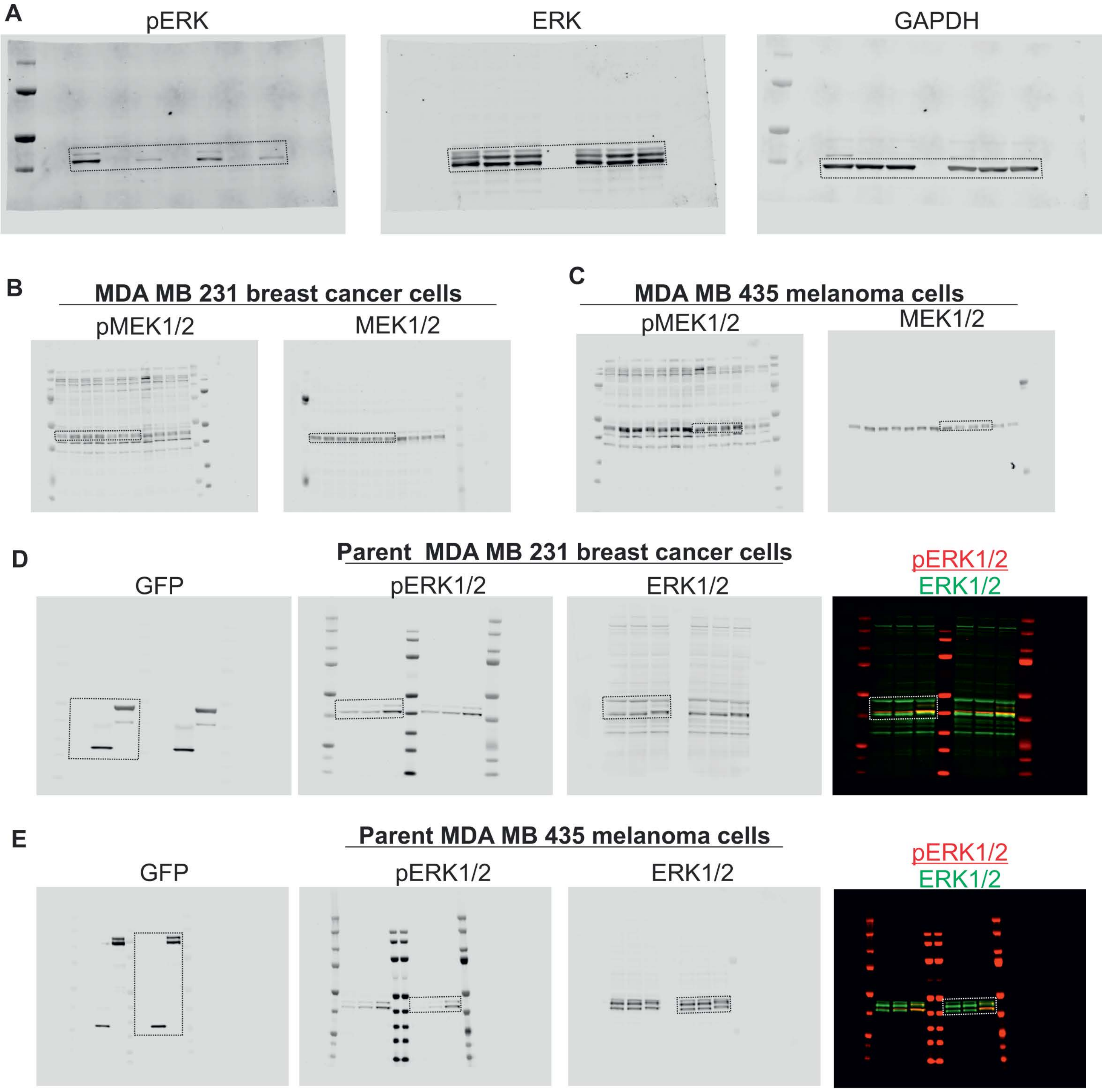
A
pERK
ERK
GAPDH
B
MDA MB 231 breast cancer cells
pMEK1/2
MEK1/2
C
MDA MB 435 melanoma cells
pMEK1/2
MEK1/2
D
Parent MDA MB 231 breast cancer cells
GFP
pERK1/2
ERK1/2
pERK1/2
ERK1/2
E
Parent MDA MB 435 melanoma cells
GFP
pERK1/2
ERK1/2
pERK1/2
ERK1/2

Fig S5. (related to Figs 4,5). **Uncropped western blots. A.** Uncropped images of phosphorylated ERK1 and ERK2 (pERK), total ERK1 and ERK 2 (ERK) and GAPDH in Sel2 MDA MB 231 and Sel1 MDA MB 435 cells that were treated with DMSO, 0.5 µM Trametinib or 10 µM U0126, with regions used to make **Fig 4D** indicated with dotted line boxes. **B.** Uncropped images of phosphorylated MEK1 and MEK2 (pMEK1/2), and total MEK1 and MEK2 (MEK1/2) in Parent, Sel and FS isolates from MDA MB 231 cells, with regions used to make **Fig 5A** indicated with dotted line boxes. **C**. Uncropped images of pMEK1/2 and total MEK1/2 in Parent and Sel isolates from MDA MB 435 cells, with regions used to make **Fig 5B** indicated with dotted line boxes. **D.** Uncropped images of GFP, phosphorylated ERK1/2, total ERK1/2 and overlay of phosphorylated (red) and total (green) ERK1/2 in transfected Parent MDA MB 231 cells, with regions used to make **Fig 7E** indicated with dotted line boxes. **E.** Uncropped images of GFP, phosphorylated ERK1/2, total ERK1/2 and overlay of phosphorylated (red) and total (green) ERK1/2 in transfected Parent MDA MB 435 cells, with regions used to make **Fig 7H** indicated with dotted line boxes.

Journal of Cell Science • Supplementary information

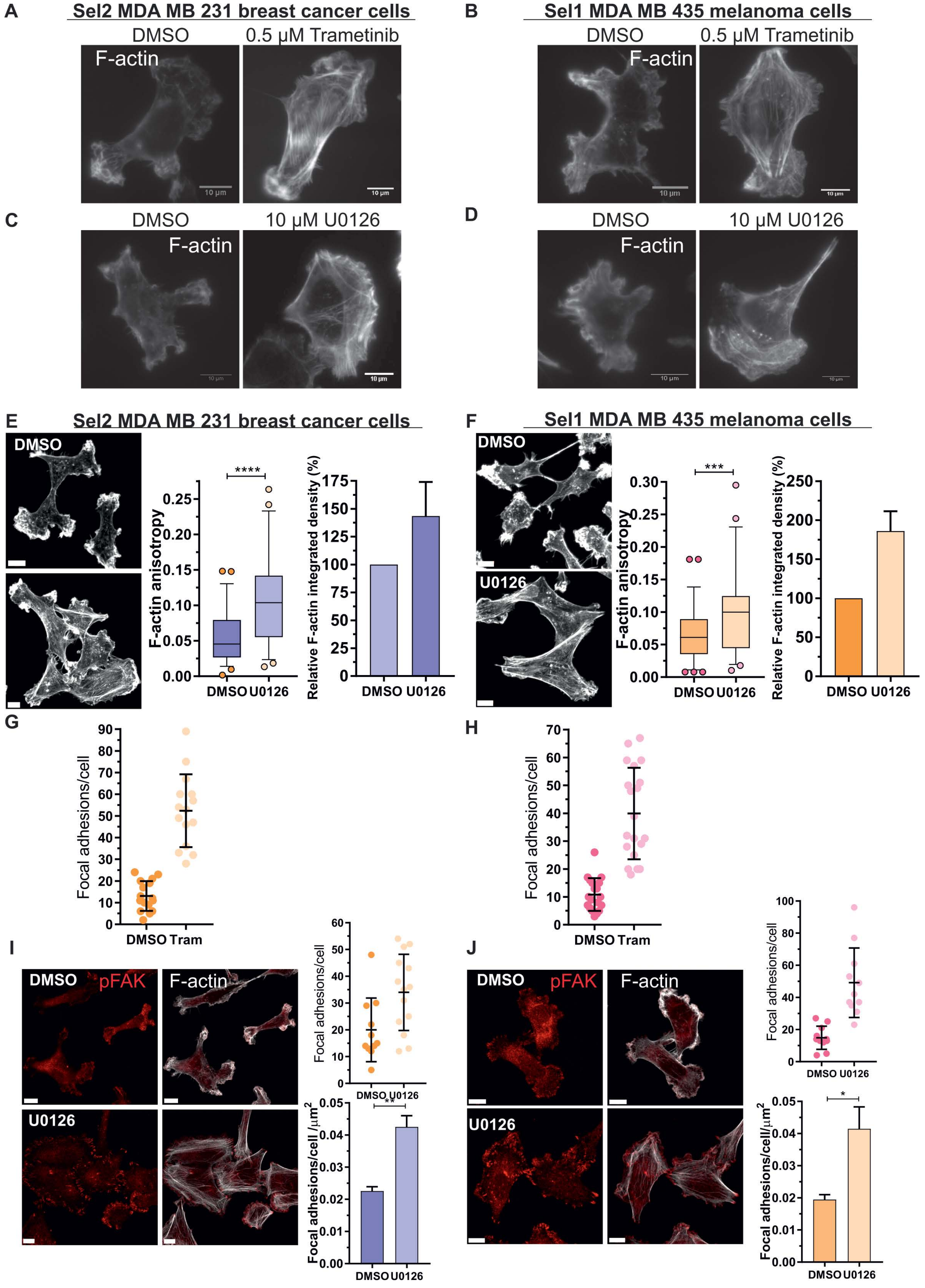

Journal of Cell Science • Supplementary information

Fig S6. (related to Fig 6) TIRF images of F-actin and focal adhesion numbers in vehicle or MEK inhibitor treated Selected MDA MB 231 and MDA MB 435 cell populations. A. TIRF microscopic images of representative Sel2 MDA MB 231 cells that had been treated with DMSO vehicle or 0.5 µM Trametinib as indicated, and then fixed and stained for F-actin structures with fluorescently-labelled phalloidin. Images were analysed with the ImageJ plugin FibrilTool [1] with corresponding results in **Fig 6A**, *middle graph;* **B.** TIRF microscopic images of representative Sel1 MDA MB 435 cells that had been treated with DMSO vehicle or 0.5 µM Trametinib as indicated, and then fixed and stained for F-actin structures with fluorescently-labelled phalloidin. Images were analysed and corresponding results presented in **Fig 6B**, *middle graph;* **C.** TIRF microscopic images of representative Sel2 MDA MB 231 cells that had been treated with DMSO vehicle or 10 µM U0126 as indicated, and then fixed and stained for F-actin structures with fluorescently-labelled phalloidin. Images were analysed with the ImageJ plugin FibrilTool [1] with corresponding results in **Fig S6E**, *middle graph*. **D.** TIRF microscopic images of representative Sel1 MDA MB 435 cells that had been treated with DMSO vehicle or 10 µM U0126 as indicated, and then fixed and stained for F-actin structures with fluorescently-labelled phalloidin. Images were analysed with the ImageJ plugin FibrilTool [1] with corresponding results in **Fig S6F**, *middle graph*. **E.** Sel2 MDA MB 231 cells were treated with DMSO or 10 µM U0126 (left panels) for 24 h, then stained for F-actin. Scale bar = 10 µm. *Middle graph;* F-actin anisotropy was scored for DMSO (n = 55) and U0126 (n = 52) treated cells. Student's t-test (**** = p<0.0001). Means ± SD. *Right graph;* Relative F-actin integrated density normalized to DMSO vehicle treated levels (100%). DMSO n = 3, U0126 n = 3 replicates. Means ± SEM**. F.** Sel1 MDA MB 435 cells were treated with DMSO or 10 µM U0126 (left panles) for 24 h, then stained for F-actin. Scale bar = 10 µm. *Middle graph;* F-actin anisotropy was scored for DMSO (n = 67) and U0126 (n = 54) treated cells. Student's t-test (*** = p<0.001). Means ± SD. *Right graph;* Relative F-actin integrated density normalized to DMSO vehicle treated levels (100%). DMSO n = 3, U0126 n = 3 replicates. Means ± SEM**. G.** Example experiment used to generate single mean focal adhesion densities in **Fig 6C**. The focal adhesion numbers per cell were determined for the following number of fixed Sel2 MDA MB 231 cells: DMSO (16), Trametinib (Tram; 15). Means ± SD. **H.** Example experiment used to generate single mean focal adhesion densities in **Fig 6D**. The focal adhesion numbers per cell were determined for the following number

Journal of Cell Science • Supplementary information

of fixed Sel1 MDA MB 435 cells: DMSO (19), Trametinib (Tram; 20). Means ± SD. **I.** *Left panels*; Immunofluorescence images showing focal adhesions stained for pFAK alone or overlayed with phalloidin-stained F-actin for fixed Sel2 MDA MB 231 cells. Scale bar = 10 µm. Upper *graph;* Example experiment used to generate single mean focal adhesion densities in **Fig S6I**, *lower graph*. The focal adhesion numbers per cell were determined for the following number of cells: DMSO (11), U0126 (14). Means ± SD. *Lower graph;* Mean focal adhesion density was determined for DMSO and U0126 treated Sel2 MDA MB 231 cells (n = 3). Student's t-test (** = p<0.01). Means ± SEM. **J.** *Left panels*; Immunofluorescence images showing focal adhesions stained for pFAK alone or overlayed with phalloidin-stained F-actin for fixed Sel1 MDA MB 435 cells. Scale bar = 10 µm. *Upper graph;* Example experiment used to generate single mean focal adhesion densities in Supplementary **Fig S6J**, *lower graph*. The focal adhesion numbers per cell were determined for the following number of cells: DMSO (11), U0126 (14). Means ± SD. *Lower graph;* Mean focal adhesion density was determined for DMSO and U0126 treated Sel1 MDA MB 435 cells (n = 3). Student's t-test (* = p<0.05). Means ± SEM.

Journal of Cell Science • Supplementary information

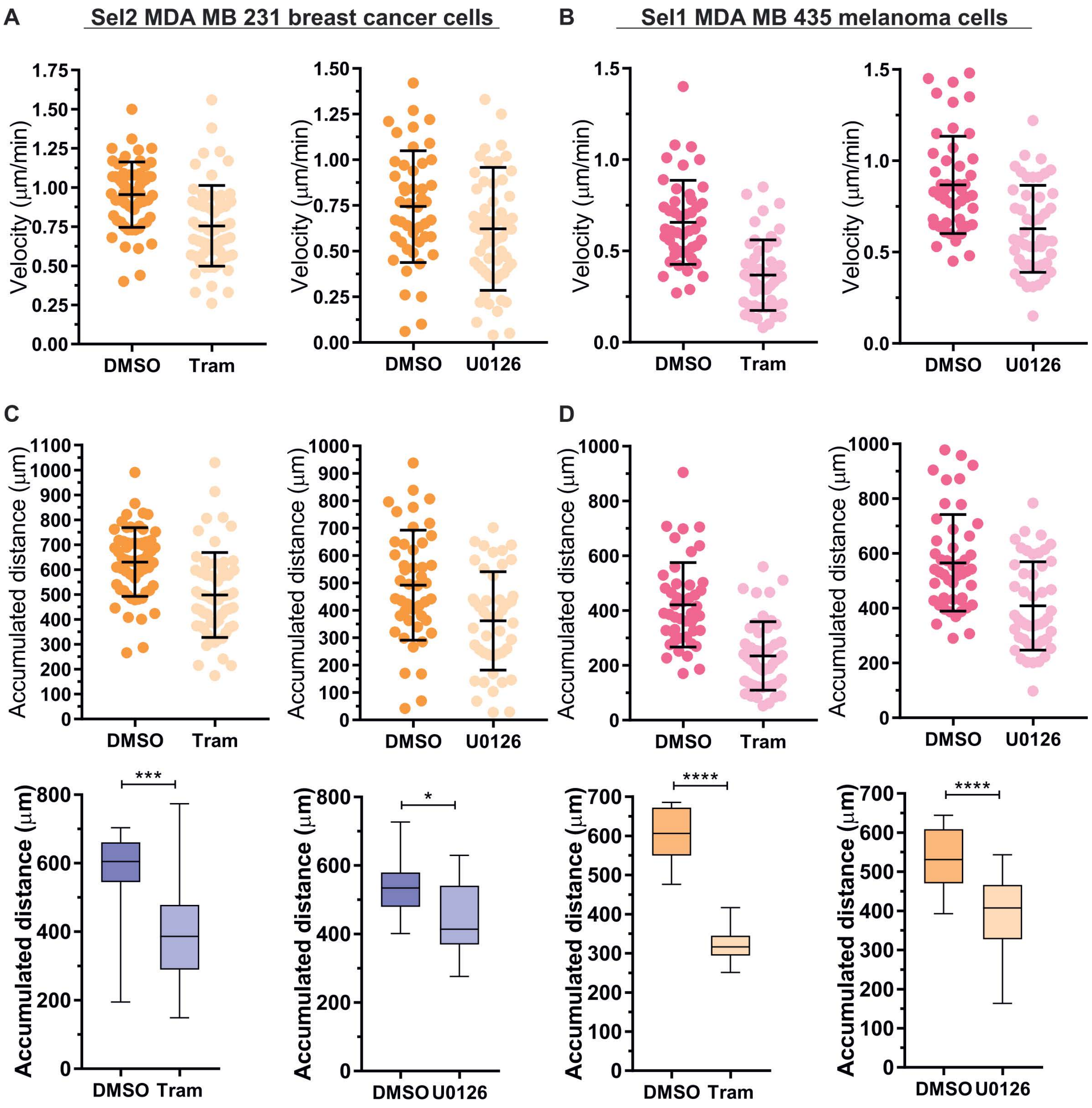

Journal of Cell Science • Supplementary information

Fig S7 (related to Fig 6) The effects of MEK inhibitors on single cell migration. A. The random migration velocities were determined for the following number of live Sel2 MDA MB 231 cells: *Left graph*; DMSO (60), Trametinib (60); *Right graph* DMSO (46), U0126 (62) as shown in **Fig 6E.** Means ± SD. **B.** The random migration velocities were determined for the following number of live Sel1 MDA MB 435 cells: *Left graph*; DMSO (46), Trametinib (48); *Right graph* DMSO (48), U0126 (48) as shown in **Fig 6F.** Means ± SD. **C.** Accumulated distances travelled by Sel2 MDA MB 231 cells as shown in **Fig S7A**. *Left panels*, DMSO or Trametinib (Tram) treatment. *Right panels*, DMSO or U0126 treatment. *Upper panels*, individual data points. *Lower panels*, Boxes indicate median, upper and lower quartiles; whiskers are 5-95% percentiles. Student's t-test. (* = $p<0.05$, *** = $p<0.001$). **D.** Accumulated distances travelled by Sel1 MDA MB 435 cells as shown in **Fig S7B**. *Left panels*, DMSO or Trametinib (Tram) treatment. *Right panels*, DMSO or U0126 treatment. *Upper panels*, individual data points. *Lower panels*, Boxes indicate median, upper and lower quartiles; whiskers are 5-95% percentiles. Student's t-test. (**** = $p<0.0001$).

Journal of Cell Science • Supplementary information

References

1. **Boudaoud, A., Burian, A., Borowska-Wykret, D., Uyttewaal, M., Wrzalik, R., Kwiatkowska, D., and Hamant, O.** (2014). FibrilTool, an ImageJ plug-in to quantify fibrillar structures in raw microscopy images. *Nat Protoc* ***9***, 457-463.

2. **Rudzka, D.A., Clark, W., Hedley, A., Kalna, G., and Olson, M.F.** (2017). Transcriptomic profiling of human breast and melanoma cells selected by migration through narrow constraints. *Sci Data* ***4***, 170172.

Journal of Cell Science • Supplementary information
